# *In Vivo* Modeling of Patient Genetic Heterogeneity Identifies New Ways to Target Cholangiocarcinoma

**DOI:** 10.1158/0008-5472.CAN-21-2556

**Published:** 2022-01-24

**Authors:** Nicholas T. Younger, Mollie L. Wilson, Anabel Martinez Lyons, Edward J. Jarman, Alison M. Meynert, Graeme R. Grimes, Konstantinos Gournopanos, Scott H. Waddell, Peter A. Tennant, David H. Wilson, Rachel V. Guest, Stephen J. Wigmore, Juan Carlos Acosta, Timothy J. Kendall, Martin S. Taylor, Duncan Sproul, Pleasantine Mill, Luke Boulter

**Affiliations:** 1 MRC Human Genetics Unit, Institute of Genetics and Cancer, University of Edinburgh, United Kingdom.; 2 Clinical Surgery, University of Edinburgh, Royal Infirmary of Edinburgh, Edinburgh, United Kingdom.; 3 Cancer Research UK Edinburgh Centre, Institute of Genetics and Cancer, Crewe Road South, Edinburgh, United Kingdom.; 4 Centre for Inflammation Research, University of Edinburgh, Edinburgh, United Kingdom.

## Abstract

Intrahepatic cholangiocarcinoma (ICC) is an aggressive malignancy of the bile ducts within the liver characterized by high levels of genetic heterogeneity. In the context of such genetic variability, determining which oncogenic mutations drive ICC growth has been difficult, and developing modes of patient stratification and targeted therapies remains challenging. Here we model the interactions between rare mutations with more common driver genes and combine *in silico* analysis of patient data with highly multiplexed *in vivo* CRISPR-spCas9 screens to perform a functional *in vivo* study into the role genetic heterogeneity plays in driving ICC. Novel tumor suppressors were uncovered, which, when lost, cooperate with the RAS oncoprotein to drive ICC growth. Focusing on a set of driver mutations that interact with KRAS to initiate aggressive, sarcomatoid-type ICC revealed that tumor growth relies on Wnt and PI3K signaling. Pharmacologic coinhibition of Wnt and PI3K *in vivo* impeded ICC growth regardless of mutational profile. Therefore, Wnt and PI3K activity should be considered as a signature by which patients can be stratified for treatment independent of tumor genotype, and inhibitors of these pathways should be levied to treat ICC.

**Significance:**

This work shows that, despite significant genetic heterogeneity, intrahepatic cholangiocarcinoma relies on a limited number of signaling pathways to grow, suggesting common therapeutic vulnerabilities across patients.

## Abstract (precis)

This work shows that, despite significant genetic heterogeneity, intrahepatic cholangiocarcinoma relies on a limited number of signaling pathways to grow, suggesting common therapeutic vulnerabilities across patients.

## Introduction

Intrahepatic cholangiocarcinomas (ICC) are epithelial tumors of the bile duct comprised of malignant ducts surrounded by an extensive stroma (1). ICC driven by infection with the liver fluke, *Opisthorchis viverrini* is endemic in South East Asia and although historically seen as a rare malignancy in the West, sporadic, nonfluke-associated disease has increased in incidence in the UK, Europe, and the United States over the last four decades. Currently, surgical resection is the only curative option for patients diagnosed with this cancer; however, of the ∼30% of patients who have disease that is amenable to surgery, 70% of those patients relapse following resection (2). In patients where surgery is not an option, the standard of care is palliative chemotherapy, which extends life by approximately 3 to 6 months (3). Early studies using either patient ICC samples (4) or mouse models (5) demonstrated that oncogenic mutations in *Kras* (typically *Kras*^G12D^) and loss-of-function mutations in *Trp53* cooperate to initiate tumor formation. Recent genomic data, however, has challenged whether mutations in this oncogene and tumor suppressor pair often co-occur in human ICC (6, 7). Instead, these sequencing data suggest that alternate or less-frequent mutations cooperate with more dominant oncogenes (such as mutant *Kras*) to promote tumorigenesis. Deep sequencing of ICC has uncovered that a high level of genetic heterogeneity exists within patient ICC samples (8, 9). Although a recurring set of mutations in canonical genes has been identified (1), many infrequent mutations have also been detected. The functional contribution of these infrequent *de novo* changes to affect disease progression and modulate therapeutic resistance or susceptibility remains unclear.

To identify and prioritize gain-of-function or loss-of-function mutations in a patient dataset of ICC, we use a computational pipeline, IntOGen (10), to generate a high-confidence list of candidate driver genes, of which, 64 have not previously been assigned as being cancer drivers. To recapitulate the clonal competition observed in human tumorigenesis, we developed an *in vivo* CRISPR-SpCas9 system, which simultaneously screens the candidate gene set against either *KRAS^G12D^* or *NRAS^G12V^* oncogenes. This identified a subset of genes in which human ICC-derived mutations genetically interact with RAS to initiate and accelerate ICC formation. Among these, we found that loss-of-function of Neurofibromin 2 (*Nf2*) interacts with mutant RAS to initiate tumor formation independent of *Trp53* status, highlighting again that as seen in patient data, RAS mutant cells do not strictly rely on *Trp53* loss to initiate ICC. Loss of *Nf2* results in the formation of aggressive and poorly differentiated sarcomatoid-type ICC. These tumors are driven by dysregulation of Wnt–PI3K signaling, highlighting a novel therapeutic avenue that could be used to target ICC growth.

## Materials and Methods

### Identification and processing of genomic data from patient datasets

#### Alignment and preprocessing of publicly available data

Exome-seq FASTQ files from Chan-on and colleagues (8) were downloaded from the European Nucleotide Archive with accession no. PRJEB4445. Exome-seq FASTQ files from Sia and colleagues (11) were downloaded from the Gene Expression Omnibus Database with accession GSE63420. The Cancer Genome Atlas (TCGA) BAM files were downloaded from the Genomic Data Commons after receiving access to individual patient BAM files. Input FASTQ files were aligned to the Hg19 reference genome. Duplicates were marked and base quality score recalibration was carried out and local indel realignment was performed. Ensemble variant calling was performed. TCGA data were input as reads which were aligned to the Hg38 reference so were re-mapped using the Hg38toHg19. IntOgen was run on each cohort individually, and then all combined. IntOgen outputs were used to build a network of known and inferred functional interactions and was clustered into modules by connection density and each module was annotated with pathway enrichments.

Evolutionary dependency (co-occurrence and mutual exclusivity) was scored using SELECT (version 1.6) with default parameters. The weighted mutual information (wMI) *P* value was used for color coding significance and FDR < 0.1 used as multitesting corrected threshold of significance. For the aggregate analysis of genes (“cosmic” and “other” groups), individual tumors were binary encoded as 1 if they contained a putative driver mutation in any gene of the corresponding gene list, and 0 otherwise.

Further experimental details about our computational approach can be can be found in Supplementary Materials and Methods.

### Design and preparation of sgRNA plasmids for *in vivo* editing

#### Generation of pooled sgRNA screening library

Oligonucleotides encoding sgRNAs targeting the set of predicted drivers were designed using spacer sequences from the mouse GeCKo V2 library (Supplementary Table S1; ref. 12). Library-specific PCR retrieval arms (13) and our schematic for library preparation is described in Supplementary Table S1. Complete sgRNA oligos for all target genes and control sequences were custom synthesized by Twist Biosciences. Purified amplicons were then digested with Esp3I, phosphorylated and ligated into the SB-CRISPR plasmid backbone.

#### Generation of single gRNAs

Single gRNAs (Supplementary Table S2; Supplementary Fig. S3A) were cloned into SB-CRISPR plasmids kindly provided by Professor Dr. Roland Rad (LMU Munich). SB-CRIPSR was digested with Esp3I or Bbsl.

### Animal work

All animal work was performed under the UK Home Office project license held by Dr. Luke Boulter (PFD31D3D4). Animals were maintained in colonies in 12-hour light–dark cycles and were allowed access to food and water *ad libitum*.

#### Hydrodynamic tail vein injection

Female, FVB/N mice from Charles River, and were used at 4 to 6 weeks of age. For the hydrodynamic tail vein injection, animals were injected with a physiologic saline solution (10% w/v) containing plasmids into the lateral tail vein. The typical injection contained 6 μg of PGK-SB13, 20 μg of CAG-Kras^G12D^ or CAG-Nras^G12V^, and 20 μg of SB-CRISPR gRNA plasmid. In models that relied on a combination of gRNA plasmids, plasmids were dissolved to a maximum concentration of 20 μg and were mixed such that they were balanced pools of each gRNA. For screening studies, the gRNA library was injected at 20 μg and we determined gRNA representation by Sanger sequencing prior to injection and plotted the GINI index for each library (Supplementary Fig. S3B).

#### *Keratin-19-CreER^T^*;*pten*^flox/flox^;*trp53*^flox/flox^;R26R^LSLtdTomato^ mice (KPPTom)

*Keratin-19-CreER^T^* mice (Jax: 026925) were crossed with animals containing floxed alleles of *Pten* (Jax: 006440) or *Trp53* (Jax: 008462) and a silenced tdTomato reporter targeted to the Rosa26 locus (Jax: 007908). All animals in this study are heterozygous for Keratin-19CreER^T^, homozygous for *Trp53^flox^* and *Pten^flox^* alleles, and homozygous for *R26R^LSLtdTomato^*. Mice received three doses of 4 mg of tamoxifen by oral gavage and followed by 400 mg thioacetamide in their drinking water. All mice were male.

### Therapeutic dosing of animal models

Animals baring *KRas^G12D^;gRNA^Nf2^;gRNA^Trp53^* tumors were randomized using GraphPad online randomization tool and dosed with either vehicle alone (10% DMSO, 40% PEG300, 5% Tween-80, and 45% saline), 5 mg/kg LGK974, 50 mg/kg pictilisib, or a combination of the two daily starting 7 days following hydrodynamic injection. KPPTom animals were given tamoxifen and thioacetamide (as detailed above) and at 4 weeks were given either vehicle alone (10% DMSO, 40% PEG300, 5% Tween-80, and 45% saline) or a combination of 5 mg/kg LGK974 and 50 mg/kg pictilisib for 4 weeks. All animals were housed in colonies of five animals.

### Isolation of RNA and DNA

Both DNA and RNA extraction used 50 to 100 mg of snap frozen tissue. DNA was extracted from tissue using the DNeasy Blood and Tissue Kit (Qiagen) as per the manufacturer's instructions. RNA was extracted using TRIzol RNA Isolation Reagent (Invitrogen), precipitated with chloroform, and cleaned up using the RNeasy Mini Kit (Qiagen). For sequencing, applications DNA and RNA quality (RIN score) was quantified using the Agilent 2100 Bioanalyzer. A minimum RIN threshold of 8 was used for RNA sequencing (RNA-seq).

### RNA-seq

Libraries were prepared from total-RNA following rRNA depletion. rRNA-depleted RNA was then DNase treated and purified prior to fragmentation Libraries were quantified by fluorometry and assessed for quality and fragment size using the Agilent Bioanalyser Sequencing was performed using the NextSeq 500/550 High-Output v2.5 (150 cycle) Kit on the NextSeq 550 platform (Illumina Inc). Libraries were combined in an equimolar pool based on Qubit and Bioanalyser assay results and run across a single High Output v2.5 Flow Cell.

### RNA-seq data processing and analysis

The primary RNA-seq processing, quality control to transcript-level quantitation, was carried out using nf-core/rnaseq v1.4.3dev (https://github.com/ameynert/rnaseq; ref. 14).

### DNA exome sequencing

Libraries were prepared from genomic DNA (gDNA) and was sheared to achieve target DNA fragment sizes of between 150 and 200bp. DNA fragments were processed as adapter-ligated libraries and were purified. Seven hundred fifty nanograms of each prepared gDNA library was hybridized to probes covering the mouse exome and hybridized DNA-probes were amplified to apply unique indexing primers. Library QC: Libraries were quantified by Qubit and sequencing was performed using the NextSeq 500/550 High-Output v2.5 (150 cycle) Kit on the NextSeq 550 platform (Illumina Inc.). Libraries were combined in a single equimolar pool and run on a High-Output v2.5 Flow Cell.

### CRISPR/Cas9-editing validation and structural variant calling

DNA sequences from exome-sequencing of tumors arising from the RAS^G12^-library screens and were aligned to the FVB mouse reference genome; subsequently, indels within 50bp upstream or downstream of sgRNA target sites were called. To determine if indels were likely due to SpCas9 activity, the interval of each indel was observed using Integrative Genomics Viewer (15), and overlain with sgRNA library binding sites. Further information on identifying the outcome of CRISPR editing is detailed in the Supplementary Materials and Methods.

### Data curation and deposition

All RNA and exome-sequencing data pertaining to this manuscript is deposited on the NCBI Gene Expression Omnibus (GEO) as accession number GSE190770.

### Histology and IHC

Livers were perfused with phosphate-buffered saline and dissected into 10% neutral-buffered formalin. Fixed tissue was processed in wax blocks and sectioned 4 μmol/L thick. Sections for immunostaining were dewaxed in xylene and rehydrated. Following antigen retrieval and sections were incubated with primary and secondary antibodies as detailed in Supplementary Table S3. Histologic assessment was undertaken by a consultant liver histopathologist working at the national liver transplant center (TJK) with experience in the comparative pathology of animal models of primary liver cancer.

### FUnGI immunostaining and clearing

We adapted a previously published protocol for FUnGI staining and imaging (16). Eight millimeters of cores were are taken from liver and cancer tissue and sectioned at 200 μm intervals using a Krumdeick Tissue Slicer and fixed in formalin. Tissues were blocked and incubated overnight with primary and secondary antibodies in a series of permeabilizing washes before being cleared in FUnGI solution, which contains high levels of fructose. Clarified tissues are incubated with DAPI and mounted in FUNGI for confocal analysis.

### Quantification of tumor burden

Histologic sections containing tumors were scanned using a Nanozoomer slide scanner with a 40× objective lens. Files were then imported into QuPath (https://qupath.github.io) and tumor tissue was manually annotated. Tumor burden represents the area of tissue occupied by tumor and number is the number of discrete tumors in the tissue. All tumor analysis was blinded.

### Reverse phase protein arrays

Snap frozen, dissected tumor tissue was provided to the Human Tumor Profiling Unit (HTPU) at the Cancer Research UK Edinburgh Centre. The target proteins analyzed by RPPA are listed in Supplementary Table S4. Reverse phase protein array (RPPA) analysis was carried out using established protocols for nitrocellulose-based arrays (17). Slide images were acquired using an InnoScan 710-IR scanner (Innopsys) with laser power and gain settings optimized for highest readout without saturation of the fluorescence signal. The relative fluorescence intensity of each array feature was quantified using Mapix software (Innopsys).

### Statistical analysis

All experimental groups were analyzed for normality using a D'Agostino–Pearson Omnibus test. Groups that were normally distributed were compared with either a two-tailed Student *t* test (for analysis of two groups) or using one-way ANOVA to compare multiple groups, with a *post hoc* correction for multiple testing. Nonparametric data were analyzed using a Wilcoxon–Mann–Whitney *U* test when comparing two groups or a Kruskal–Wallis test when comparing multiple nonparametric data. Throughout, *P* < 0.05 was considered significant. Data are represented as mean with SEM for parametric data or median with SD for nonparametric data.

All figures were laid out with Adobe Illustrator and graphics were created with BioRender.com.

### Ethics approval and consent to participate

All human data included in this manuscript comes from previously published, consented studies. Animal work performed here is approved by the UK Home Office license provided to L. Boulter.

### Availability of supporting data

All -omics data are deposited in publicly accessible databases, and all other datasets used and analyzed during the current study are available within the manuscript and its additional files.

## Results

### Identifying candidate causative mutations that drive ICC growth

ICC contains a range of infrequently mutated genes without known function. Identifying a consensus group of driver mutations in ICC using exome and genome sequencing has been challenging, due in a large part to tissue availability. Nonetheless, a number of studies have demonstrated recurrent ICC mutations including neomorphic alterations in *IDH1* and *IDH2*, loss-of-function mutations in *PBRM1*, *BAP1*, *TP53*, *ARID1A*, and gain-of-function mutations in *KRAS*. Despite their identification, the presence of these mutations in a tumor is not a strong predictor of therapeutic outcome (18, 19) and for approximately 30% of patients with ICC a driver mutation cannot be identified (20).

**Figure 1.**
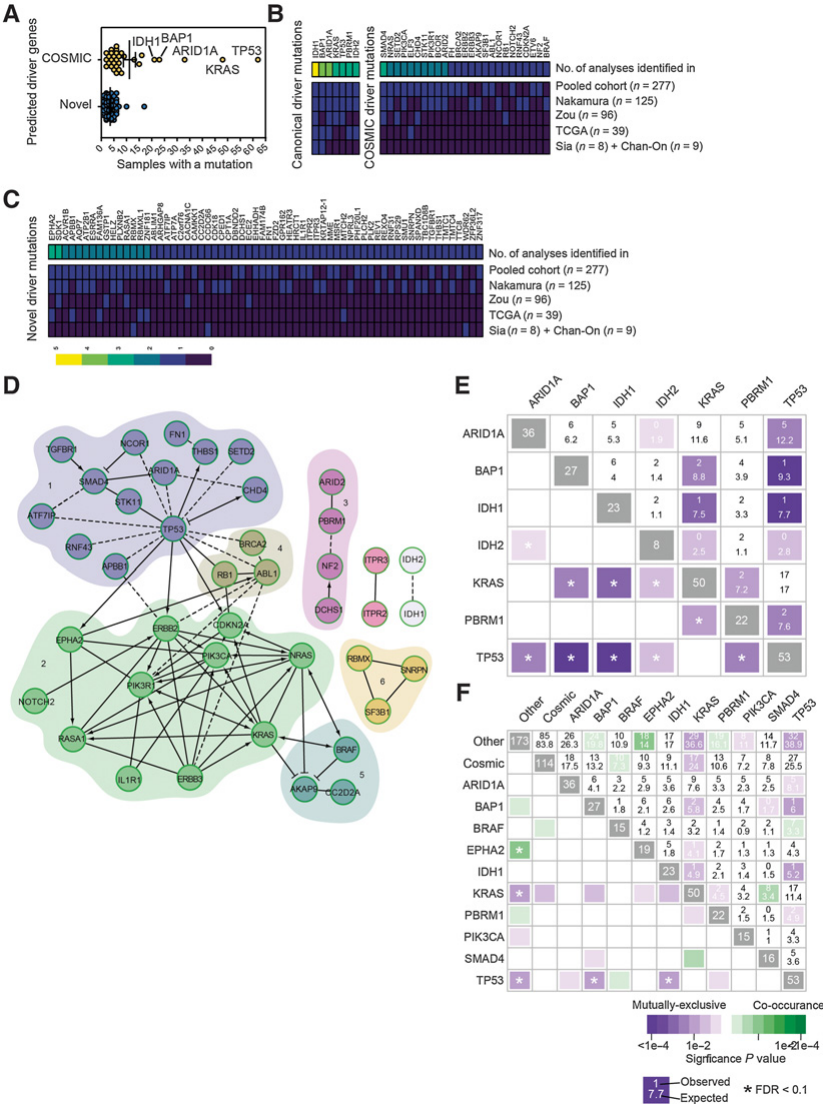
*In silico* screening identifies novel drivers of ICC. **A,** The number of samples with mutations in driver genes identified following analysis with IntOGen. Samples clustered into those that had been previously identified in ICC or are present in the COSMIC database (yellow points), or those mutated genes that have not previously been assigned as being cancer drivers (blue points). **B,** Frequency of samples containing known ICC or COSMIC mutation in each of the individual cohorts collated in this study and in the pooled datasets. **C,** Frequency of samples containing a predicted novel oncogenic mutation identified by IntOGen. In both **B** and **C,** heatmaps represent the frequency at which each mutation is found within each study and the aggregated frequency between all studies. Top bar represents the number of times a mutation was identified between studies. **D,** Pathway interaction analysis of putative ICC driver mutations identified by IntOGen based on known functional (solid lines) and predicted physical (dotted lines) interactions. Numbers 1 to 6 represent distinct gene relationship modules based on predicted or known genetic interactions. **E,** Co-occurrence and mutual exclusivity analysis demonstrates that there is a high level of mutational exclusivity between canonical driver mutations in patients with ICC. **F,** Co-occurrence and mutual exclusivity of COSMIC drivers that co-occur ≥15 times in the human sample set and combined “other,” which includes all novel drivers. *N* = 277 patient exomes or genomes with matched, noncancerous tissue.

To determine whether all patient tumors contain potential driver mutations, we used a computational pipeline based around the driver prediction tool IntOGen (10). This method utilizes a combination of functional impact bias (OncodriveFM), spatial clustering (OncodriveCLUST), and corrected frequency (MutSigCV) to define whether particular genomic regions have a mutational rate beyond that which is expected, have a bias towards clustered mutations or those that are likely to impact functional domains, such as those that are regulatory or catalytic (summarized in Supplementary Fig. S1). Having filtered out hypermutated samples (Supplementary Table S1), we used this pipeline to analyze the variants identified in the genomes of 277 sporadic and fluke-associated ICCs from four distinct studies (7, 11, 21, 22), summary information on the aggregated cohort can be found in Supplementary Figs. S2A to S2H and Supplementary Table S2. Following processing, 55% of samples (*N* = 152) carried ≥2 predicted drivers whereas 18% (*N* = 50) carried none (Fig. 1A; Supplementary Table S3). Of these predicted driver mutations, approximately one third of mutations were already known to occur in ICC or were present in genes in the COSMIC database (Fig. 1B). The remaining two thirds of mutations were novel and occurred in fewer than 8% of ICC cases. Indeed, the majority of novel mutations were only found in three to four patients, corresponding to ∼1.5% of the patient cohort (Fig. 1C; Supplementary Tables S4 and S5). To explore whether these low-frequency predicted drivers are involved in common pathways or processes, networks were constructed on the basis of known and predicted physical interactions (23) and clustered into modules based on connection density (Fig. 1D; ref. 24). This produced a network with six modules containing a mix of known in ICC, COSMIC, and novel genes. Gene ontology analysis was performed on the modules to ascertain the biological processes in which each module may participate (Supplementary Table S6). Finally, we sought to determine whether, based on our mutations as defined through IntOgen, we could define the genetic interactions between mutations in this patient dataset. Within our group of canonical driver mutations, we found that (with the exception of *KRAS* and *TP53*) there is a consistent and high level of mutual exclusivity between canonical driver genes, suggesting that the recurrent mutations found across studies are not interdependent for ICC initiation and growth (Fig. 1E). Interestingly, when we expanded this analysis to include COSMIC mutations and novel mutations (those that recur ≥15 times within our dataset *BRAF*, *PIK3CA*, *EPHA2*, and *SMAD4* are individually identified and those with ≤15 occurrences within this set are grouped as “cosmic” and novel are defined as “other”), we found distinct patterns of co-occurrence and mutual exclusivity that support the idea that there are *bona fide* genetic interactions between canonical driver genes (e.g., *KRAS*) and the large group of novel, infrequently mutated genes identified by IntOgen (Fig. 1F).

### *In vivo* CRISPR-SpCas9 screening identifies novel tumor suppressors in RAS-driven ICC

Clonal analysis of patient with ICC has failed to identify a consensus mutational route through which tumors progress (25) or epistatic mutations that functionally interact to drive tumor initiation and growth. Relatively low sample number and high genetic heterogeneity in ICC exacerbate the difficulties with this type of associative analysis.

**Figure 2.**
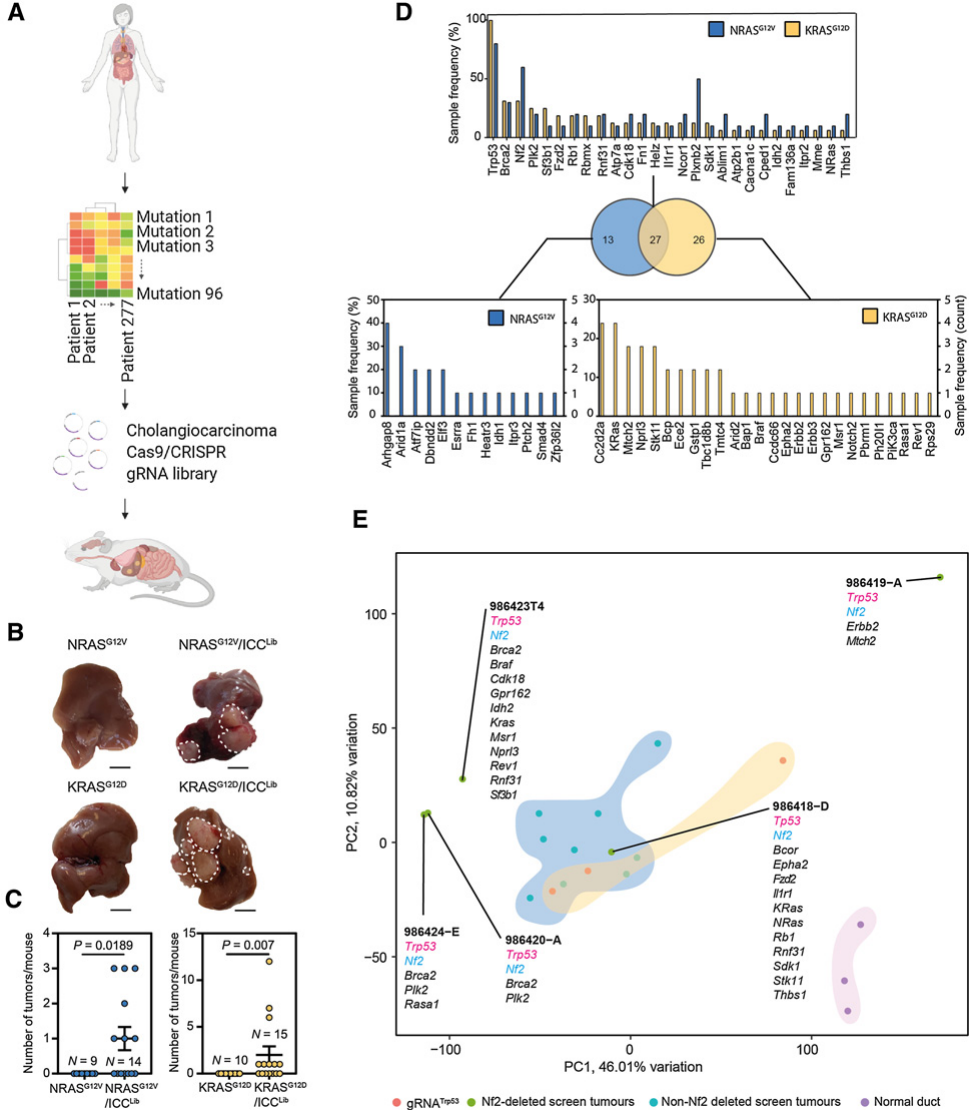
*In vivo* CRISPR-Cas9 screening identifies transforming mutations that interact with mutant Ras. **A,** Schematic of this study in which high-content sequencing data are collated from patients with ICC and the mutational profile of these tumors rationalized to identify novel, high confidence drivers of ICC. These putative drivers are used as input for an *in vivo* SpCas9/CRISPR screen to identify novel functional processes that drive ICC growth. **B,** Macroscopic images of the livers following injection with either NRAS^G12V^ or KRAS^G12D^ alone (left) or in combination with ICC^Lib^ (right; dotted line, tumor). Scale bar, 1 cm. **C,** Quantification of macroscopic tumors per mouse at 10 weeks in mice bearing Nras^G12V^ -expressing tumors and 8 weeks in those with Kras^G12D^-driven cancer. Each circle represents a different animal. **D,** The number of samples containing Indels in a particular gene following whole exome sequencing. Top graph lists those mutations found in both NRAS^G12V^ and KRAS^G12D^ tumors, and bottom graphs denote those mutations that are found only in KRAS^G12D^ - or NRAS^G12V^ -expressing tumors. Sample frequency (%) denotes the proportion of tumors containing any given mutation, whereas (count) is absolute number. (*N* represents anatomically discreet tumors recovered from at least four individual animals, KRAS^G12D^*N* = 14 and NRAS^G12V^*N* = 10.) **E,** PCA showing how samples group based on their transcriptomic signature and gRNA-induced mutations associated with each tumor type.

To overcome these limitations and to define which candidate drivers are functionally capable of initiating ICC, we developed an *in vivo* screening approach that allowed us to functionally prioritize ICC driver mutations (Fig. 2A). Previous work using multiplex-mutagenesis in the liver has demonstrated that editing specific genomic loci in hepatocytes can give rise to ICC, albeit using a relatively limited pool of gRNAs, targeting 10 genes (26). In this system, naked DNA is delivered to the liver using a high pressure, hydrodynamic injection into the lateral tail-vein of mice, an approach that has been used in the past to deliver cDNAs coding oncogenes (27, 28). When paired with CRISPR-SB plasmids containing sgRNAs and SpCas9 flanked between two Sleeping Beauty (SB) inverted terminal repeats, and a plasmid expressing Sleeping Beauty (SB) transposase, it is possible to edit *in vivo* endogenous genes in hepatocytes in a mosaic manner (29). Using the CRISPR-SB system as a starting point, we generated a large-scale multiplexed CRISPR-SpCas9 plasmid library (known hereafter as ICC^Lib^) containing triplicate gRNAs targeting 91 mouse homologues of our putative, patient-derived ICC driver genes identified through our *in silico* approach. Five genes (*KRTAP12–1*, *RBMXL1*, *RBMX*, *SPANXD*, and *ZNF181*) from our human dataset had no identifiable murine orthologue, and so were excluded from further analysis (Supplementary Figs. S3A and S3B).

Using this CRISPR-SpCas9 system, we randomly introduced CRISPR-SpCas9 targeted mutations into these candidate “patient-led” ICC genes in otherwise wild-type mice to determine which loss-of-function mutations are necessary for tumor initiation and determine whether any loss-of-function mutations interact to drive ICC formation. The ICC^Lib^ alone failed to induce any tumors in mice after 10 weeks, suggesting that within this timeframe these loss-of-function mutations alone are insufficient to initiate cancer. Gain-of-function mutations in *KRAS* and *NRAS* have been previously described in ICC (8) and through our IntOGen analysis, we similarly identified recurrent mutations in both of these genes (*KRAS* 18.05% and *NRAS* 2.88%, Supplementary Table S4). In experimental models, expression of mutant RAS in the adult liver is weakly oncogenic and normally insufficient to initiate ICC formation; rather, mutant cells undergo oncogene-induced senescence and are removed from the liver by immune clearance (30). Therefore, we co-expressed cDNAs of GFP tagged KRAS^G12D^ or NRAS^G12V^ with our ICC^Lib^ to determine whether any of the loss-of-function mutations introduced via the ICC^Lib^ synergize with mutant RAS proteins to promote ICC initiation. Within 10 weeks, mice that received either KRAS^G12D^ or NRAS^G12V^ and loss-of-function mutations induced by the CRISPR-SpCas9 containing ICC^Lib^ developed macroscopic and multifocal cancer; this was accelerated in KRAS^G12D^ mice, which developed symptomatic liver cancer in 8 weeks (Fig. 2B). In those mice that developed cancer, multiple tumors formed per mouse (Fig. 2C), which were histologically aggressive adenocarcinoma with a poorly differentiated cholangiocellular morphology. Importantly, these tumors express GFP (Supplementary Fig. S4A), denoting that they continue to express the KRAS^G12D^ or NRAS^G12V^ constructs, and the cholangiocyte marker Keratin-19, which is constrained to the biliary epithelium in normal livers (Supplementary Fig. S4B). Together, these data demonstrate that mutant Ras (NRAS^G12V^ or KRAS^G12D^) can interact with at least one loss-of-function mutation generated by the gRNAs contain in the ICC^Lib^ to initiate ICC *in vivo*.

To determine which CRISPR-SpCas9 mutational events cooperated with KRAS^G12D^ or NRAS^G12V^ and lead to the emergence of liver cancer, whole exomes were sequenced from 14 KRAS^G12D^ and 10 NRAS^G12V^ driven tumors. All indels within 50bp of a sgRNA target site were manually inspected to determine whether they were Cas9-induced or of spontaneous origin. Almost all indels had start or end positions approximately 3bp upstream of the SpCas9 protospacer adjacent motif (PAM) sequence, strongly indicating that they are a consequence of CRISPR-SpCas9 editing (Supplementary Fig. S5A). Our data shows that tumors acquired multiple CRISPR-SpCas9-induced lesions; KRAS^G12D^ tumors contained an average of 7.5 ± 1.19 mutations and in NRAS^G12V^ tumors there were on average 7.7 ± 2.59 CRISPR-SpCas9 induced mutations (Supplementary Fig. S5B). Across both Kras^G12D^ and Nras^G12V^ screens, 66 of the 91 predicted drivers targeted with the ICC^Lib^ were mutated and 27 of these were shared between NRAS^G12V^ and KRAS^G12D^ tumors (Fig. 2D; Supplementary Fig. S6). The most common CRISPR-SpCas9 mutation we identified was unsurprisingly in *Trp53*, reiterating the ability of cells with *Trp53* loss-of-function mutations to overcome RAS-induced senescence (31). We also found recurrent CRISPR-induced indels from both NRAS^G12V^ and KRAS^G12D^ screens in genes whose loss has been linked to ICC but that have not previously been shown to genetically interact with mutant RAS in this cancer including *Brca2*, *Nf2*, and *Plk2* (Fig. 2D).

### *Nf2* loss interacts with KRAS^G12D^ and *Trp53* loss to promote sarcomatoid phenotypes in ICC

**Figure 3.**
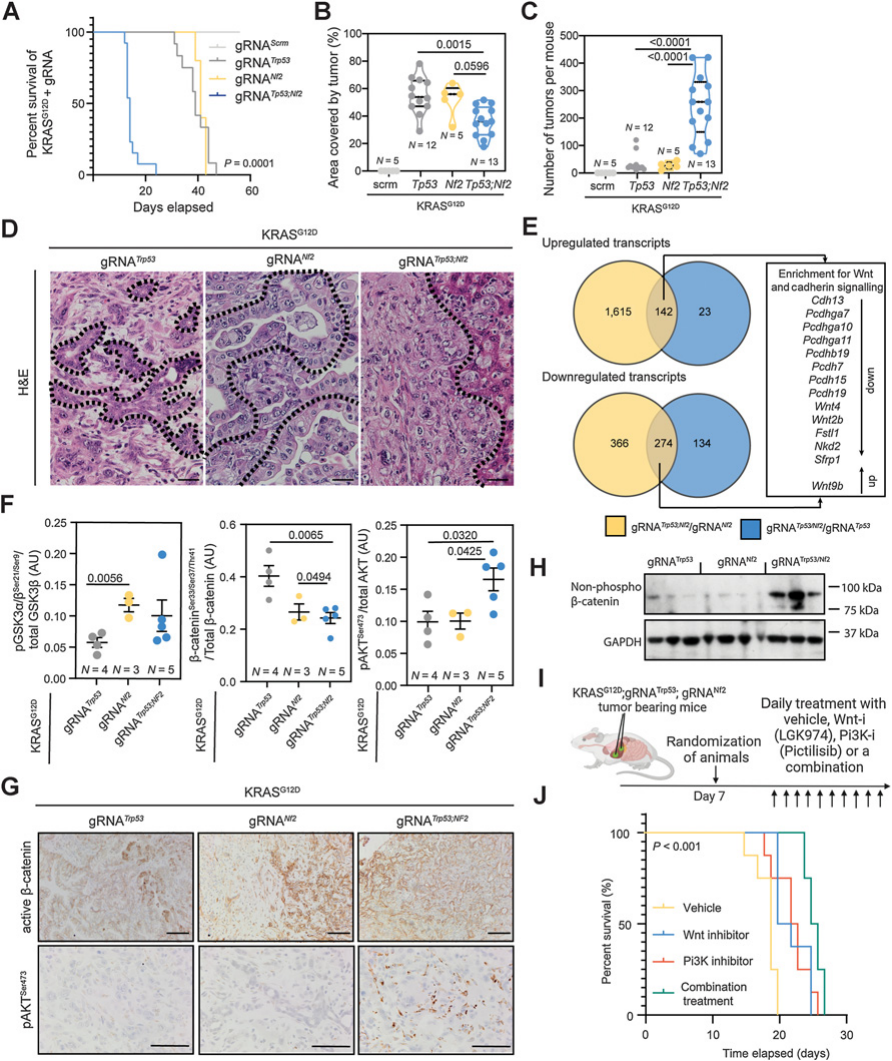
*Nf2* loss results in Ras^G12D^-induced oncogenesis and cooperates with *Trp53* loss to accelerate ICC formation. **A,** Kaplan–Meier curve demonstrating the relative survival proportions of mice with KRAS^G12D^ and gRNAs targeting *Trp53* (*N* = 12), *Nf2* (*N* = 5), *Nf2*;*Trp53* (*N* = 13), or nontargeting control (scrm, *N* = 5). **B** and **C,** Proportion of liver occupied by tumor (**B**) and number of tumors per mouse (**C**). **D,** Hematoxylin and eosin (H&E) staining of KRAS^G12D^ tumors with *Trp53*, *Nf2*, or *Trp53*;*Nf2* loss. Scale bar, 100 μm. Dotted line, tumor-stroma boundary. **E,** Comparison of RNA-seq analysis when the transcriptomes from *Nf2*;*Trp53* versus *Trp53* alone tumors (blue) are compared with transcripts from *Nf2*;*Trp53* versus *Nf2* alone (yellow) tumors. Each group contains *N* = 4 regionally distinct tumors. **F,** Analysis of RPPA data demonstrating the changes in the proportion of phosphorylated GSK3α/β, β-catenin, and pAKT relative to total protein levels in KRAS^G12D^;*Trp53*^KO^ (gray points), KRAS^G12D^;*Nf2*^KO^ (yellow points), KRAS^G12D^;*Trp53*^KO^;*Nf2*^KO^ (blue points). **G,** IHC of active, dephosphorylated β-catenin (top) and phosphorylated AKT^Ser647^ (bottom) in KRAS^G12D^;*Trp53*^KO^, KRAS^G12D^;*Nf2*^KO^, KRAS^G12D^;*Trp53*^KO^/*Nf2*^KO^ tumors. Scale bar, 50 μm. **H.** Immunoblot for dephosphorylated (active) β-catenin (β-catenin^Ser33/37/Thr41^) in tumors isolated from mice baring Kras^G12D^ -driven ICC with *Trp53*, *Nf2*, or *Trp53*;*Nf2* co-loss. GAPDH was used as a loading control. **I,** Schematic representing our dosing approach to determine whether Wnt inhibition, PI3K inhibition, or a combination of the two is effective in improving the survival of mice with KRAS^G12D^;*Trp53*^KO^;*Nf2*^KO^ ICC. **J,** Kaplan–Meier curve demonstrating the survival changes when KRAS^G12D^;*Trp53*^KO^;*Nf2*^KO^ animals are treated with vehicle (yellow line), LGK974 (Wnt-inhibitor; blue line), pictilisib (PI3K inhibitor; orange line), or a combination (green line; *N* = 5 per group).

Our data demonstrate that loss of numerous genes mutated at low frequency in human ICC potentially interact with activating NRAS^G12V^ and KRAS^G12D^ mutations to promote ICC initiation *in vivo*. These data and those of others has demonstrated that *KRAS* mutations occur more frequently in ICC than those in *NRAS* (9), therefore we prioritized validating loss-of-function mutations that genetically interact with KRAS^G12D^. From our screen, we identified which mutations in patients have the potential to initiate the formation of Ras-induced ICC, therefore we set about to define whether any particular mutation (or set of mutations) alters the phenotype of these cancers. RNA sequencing analysis of our ICC^Lib^ screen tumors showed that, on the whole, tumors transcriptionally clustered closely to each other by principal component analysis (PCA) and were transcriptionally similar to tumors generated by expressing both KRAS^G12D^ and deleting *Trp53* (Fig. 2E). Furthermore, our screen tumors were transcriptionally distinct from normal bile ducts. However four tumors in our screen transcriptionally segregated from all other tumors; all contained SpCas9/CRISPR-induced mutations in both *Trp53* and *Nf2*. In fact, of the 14 cancers from our screen that we exome sequenced, four of the five containing *Nf2* mutations segregated away from the main cluster (Fig. 2E), suggesting that the addition of a mutation in *Nf2* can functionally cooperate with *Kras^G12D^* and *Trp53* mutations and affects the phenotype of ICC. NF2 is also known as Merlin and has a well-defined role in the Hippo/LATS signaling pathway, where it negatively regulates pathway activation through the phosphorylation of *Mst1/2*; however, NF2/Merlin is also known to interact with a number of other signaling pathways including PI3K and Wnt signaling (32). We elected to investigate the genetic interaction of *Nf*2, *Trp53*, and *Kras^G12D^* further, by generating gRNAs to specifically target and disrupt the *Nf2* and *Trp53* loci (or a nontargeting control, gRNA^scrm^), which were then coinjected hydrodynamically with our KRAS^G12D^ expressing construct to define whether loss of these tumor suppressors can specifically cooperate with KRAS^G12D^ to promote tumor initiation. *Trp53* loss and *Nf2* loss were both capable of overcoming the senescence inducing effects of Kras^G12D^ expression in the liver and mice developed lethal tumors within 8 weeks following injection (Fig. 3A). In the presence of KRAS^G12D^, the singular deletion of either *Trp53* or *Nf2* resulted in large discrete tumors. However, dual loss of *Trp53* and *Nf2* resulted in cancers that were highly aggressive and invasive, which had a median survival of 14 days compared with 39 and 41 days in singular *Nf2*-deleted and *Trp53*-deleted tumors, respectively (Fig. 3A). Tumors that lacked both *Trp53* and *Nf2* were highly diffuse and covered less liver area compared with both single gene deletions; however, the number of tumors that formed was significantly higher in *Trp53*;*Nf2* codeleted tumors, suggesting that mutations in these two tumor suppressors may synergize in cancer and are not functionally redundant (Fig. 3B and C). Rather, the codeletion of *Trp53* and *Nf2* enables the increased retention of mutant Kras^G12D^-expressing cells within the liver. This retention leads to more cancers forming and that the accelerated mortality seen in animals bearing *Trp53*;*Nf2* codeleted tumors is likely due to the number of tumors that are able to form, rather than due to their size. Histopathologically, the *Trp53*;*Nf2* codeleted cancers are highly invasive with CK19 and GFP (KRas^G12D^-expressing) immunopositive cells migrating throughout the liver (Fig. 3D; Supplementary Fig. S7) and represent a model to study the biology of invasive, sarcomatoid ICCs, which migrate along the ducts and invade the liver (33). Rather than those ICCs, which are mass forming (and which have been previously modeled in mice), sarcomatoid ICC, although rare, has a very poor prognosis with a survival of weeks to months following diagnosis (34). In mouse, these *Trp53*;*Nf2* codeleted sarcomatoid cancers grow rapidly, however, we did not observe perineural or microvascular invasion, nor did we see enlarged lymph nodes in these mice, suggesting the main cause of lethality is loss of functional liver capacity rather than the exit of cells from the tumor mass.

To determine the transcriptomic differences driving this aggressive phenotype in *Trp53*;*Nf2* codeleted cancers when compared with individual *Trp53* or *Nf2* deleted tumors, we undertook bulk RNA-seq of tumors with Kras^G12D^;*Trp53*^KO^, Kras^G12D^;*Nf2*^KO^ or Kras^G12D^;*Trp53*^KO^;*Nf2*^KO^ genetic profiles. Sarcomatoid, *Trp53*;*Nf2* codeleted tumors are transcriptionally distinct from cancers containing either *Trp53* or *Nf2* deletions alone based on PCA (Supplementary Fig. S8A; Supplementary Table S7). By comparing the up- and downregulated genes in *Trp53* versus *Trp53*;*Nf2* codeleted tumors against the changes found in *Nf2* versus *Trp53*;*Nf2* codeleted tumors, we identified enrichment in signatures for Wnt signaling and Cadherin signaling using PANTHER (Supplementary Fig. S8B) in our model of sarcomatoid ICC. However, we did not find a transcriptional signature for Hippo signaling (which we would anticipate based on NF2′s canonical role in this pathway), nor could we identify YAP-positive cells within the *Nf2*-deleted tumors, rather YAP-positive cells are only found adjacent to the tumor mass. Together, these data indicate that *Nf2* loss in these tumors fails to activate Hippo signaling (Fig. 3E; Supplementary Figs. S8B and S8C). Previous work from our group has shown that Wnt signaling promotes ICC growth in the absence of classical Wnt pathway “activating mutations” (35). In *Trp53*;*Nf2* codeleted cancers, we observed upregulation of *Wnt9b* and suppression of inhibitors of Wnt signaling *Nkd2* and *Sfrp2*, suggesting that alterations in ligand levels and negative regulators of Wnt signaling are important mediators of ICC progression and that these cancers form independent of core Wnt-activating mutations in *Apc*, *Axin2*, and *Ctnnb1* (36). Interestingly, sarcomatoid ICC in patients displays changes in cadherin expression, although it is not clear whether these changes are causative for the aggressive sarcomatoid phenotype (37). In our model, we found transcriptional suppression of a range of cadherins and proto-cadherins that have been also implicated in Wnt regulation (38).

As there are few targeted treatments for poorly differentiated ICC, we screened our models driven by KRAS^G12D^-expression and either *Tp53* loss, *Nf2* loss, or a combination of the two for activated and pharmacologically targetable signaling pathways using highly multiplexed RPPAs. Deletion of *Nf2* (either alone or in combination with *Trp53*) results in increased inhibitory phosphorylation of GSK3β and activation of β-catenin signaling when compared with KRAS^G12D^ driven tumors lacking *Trp53* alone. These changes demonstrate that in *Nf2-mutant* cancers the canonical Wnt signaling pathway is highly activated and that the alterations in levels of Wnt ligands and inhibitors found at the transcriptional level translate into increased pathway activation (Fig. 3F). Furthermore, when *Trp53* and *Nf2* are concurrently deleted, the proportion of AKT that is phosphorylated at Serine-473 significantly increases (Fig. 3G), indicating that within these Kras^G12D^;*Trp53*^KO^;*Nf2*^KO^ cancers signaling via AKT is also elevated. Histologically, β-catenin is found within all KRAS-driven cancers (Fig. 3G) but the dephosphorylated (active) form of the protein is increased in protein lysates from Kras^G12D^;*Trp53^KO^*;*Nf2^KO^* tumors (Fig. 3H). These data suggest that concurrent pAKT and Wnt activity promote the development of sarcomatoid ICC and that this combination of mutations is also sufficient to suppress apoptosis in these cancer cells (Supplementary Figs. S9A and S9B), thereby promoting the development of cancer in the liver, which rapidly becomes lethal. To test whether the aggressiveness of *Trp53*;*Nf2* mutated ICC is dependent on Wnt and AKT signaling directly, we treated mice bearing KRAS^G12D^;*Nf2*;*Trp53*-KO tumors with an inhibitor of Porcupine (LGK974), which reduces Wnt ligand secretion by preventing palmitoylation of Wnt ligands and a PI3K inhibitor, pictilisib, which prevents the conversion of PIP_2_ into PIP_3_ and thereby reduces AKT phosphorylation. Animals were given sarcomatoid ICC (by a hydrodynamic injection of KRAS^G12D^;gRNA*^Nf2^*;gRNA*^Trp53^*) and at 7 days following tumor initiation were randomized to receive vehicle, LGK974, pictilisib or a combination of the two compounds (combination treatment), summarized in Fig. 3I. Both LGK974 and pictilisib significantly improve survival of tumor bearing mice compared with animals treated with vehicle alone [median survival of 19 days in vehicle treated vs. 21 days in LGK974-treated (*P* = 0.0015) and 22.5 days in pictilisib-treated animals (*P* = 0.0058), respectively]. Wnt inhibition and PI3K inhibition performed similarly, with no statistically significant difference in survival outcomes when used as single agents (Fig. 3J; Supplementary Figs. S10A–S10C; Supplementary Table S8). In combination, LGK974 and pictilisib improve median survival from 19 to 25.5 days (Fig. 3J), significantly (*P* < 0.001) reducing mortality compared with single treatments (Supplementary Figs. S10D–S10F) and demonstrating that coinhibition of Wnt and PI3K signaling is an effective treatment in sarcomatoid tumors that lack classical Wnt and PI3K activating mutations (i.e., mutations in *APC*, *CTNNB1*, and *PI3KCA*).

### Wnt and PI3K/AKT represent a conserved mechanism by which distinct pathologic subtypes of ICC grow

**Figure 4.**
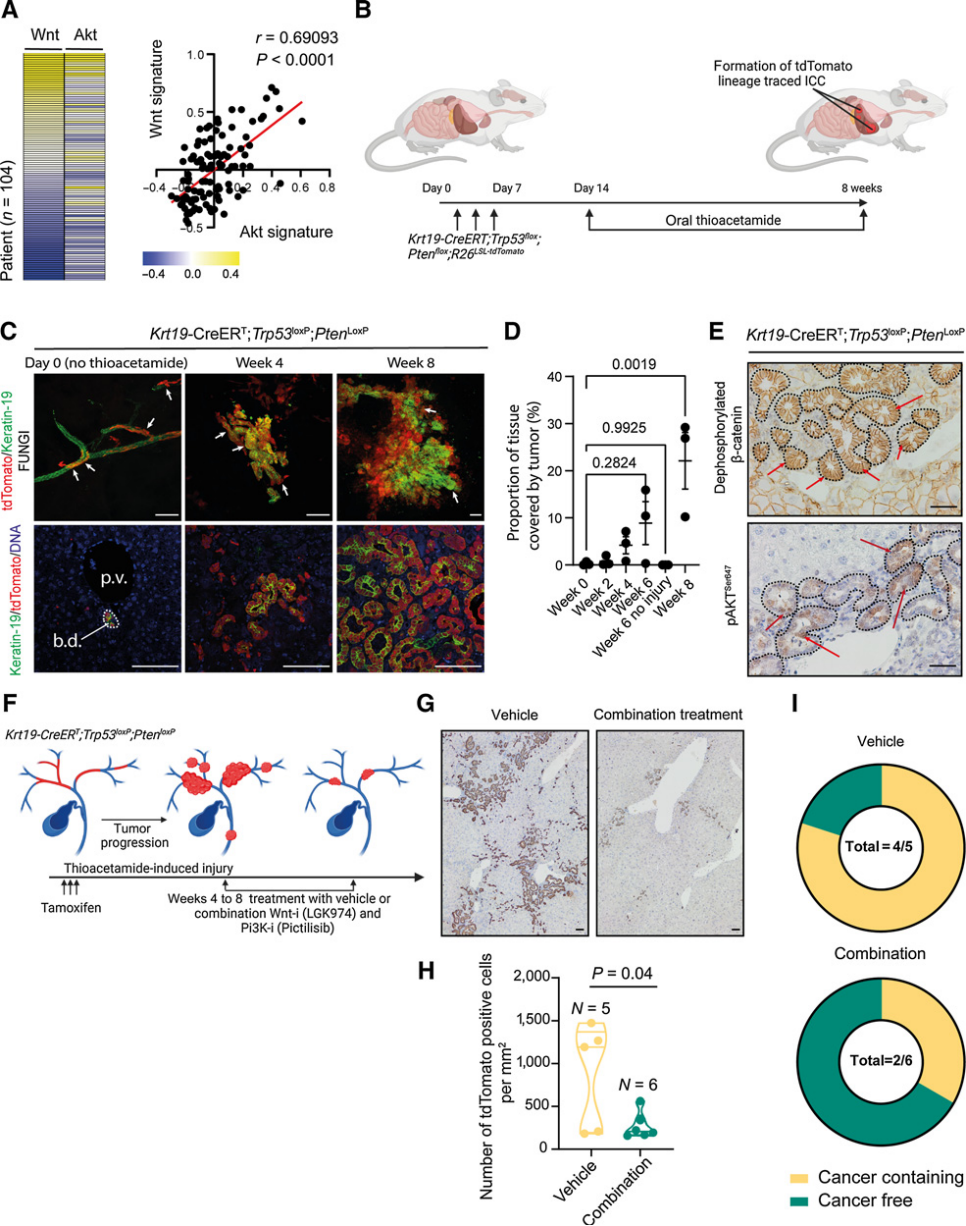
Therapeutic coinhibition of Wnt and PI3K signaling reduces tumor growth in ICC. **A,** RNA-seq data of human ICC demonstrating a positive correlation between the activity of canonical Wnt signaling and Akt signaling. **B,** Schematic representation of the KPPTom cholangiocarcinoma model where Cre^ERT^ expression in Keratin-19–positive cholangiocytes results in the inactivation of *Trp53* and *Pten*, whereas labeling transformed cells with tdTomato. **C,** Representative IHC staining of KPPTom model following tamoxifen administration (day 0) and following 4 and 8 weeks of thioacetamide administration. tdTomato (red) denotes recombined cholangiocytes (denoted by Keratin-19; green). Blue, DNA. Top, whole mount FUNGI images; bottom, 2D histologic sections. Scale bar, 200 μm. White arrows, tdTomato-positive cells. **D,** Quantification of liver tissue occupied by tumor in the KPPTom ICC model. **E,** IHC showing that KPPTom ICC has activated canonical Wnt signaling [by staining for dephosphorylated (active) β-catenin] and PI3K activity (through pAKT^Ser647^ positivity). Red arrows, positive cells. Scale bar, 100 μm. **F,** A schematic representation of how the KPPTom model was used to test the effectiveness of Wnt and Pi3K inhibitor combinations on ICC progression. **G,** IHC staining for tdTomato-positive cancer cells in vehicle-treated animals compared with those treated with a combination of LGK974 and pictilisib. Scale bar, 100 μm. **H,** Number of tdTomato-positive cells in KPPTom animals given vehicle or LGK974 and pictilisib in combination**. I,** Proportion of KPPTom animals containing macroscopic tumors in KPPTom animals treated with vehicle versus combination treatment. b.d., bile duct; p.v., portal vein.

From previous transcriptomic studies of ICC, it is possible to identify a subgroup of patients with high Notch pathway activity and who would be sensitive to treatment with γ-secretase-inhibitors (39). We therefore sought to identify whether there is a group of patients with ICC who could be sensitive to coinhibition of Wnt and PI3K signaling. In 104 transcriptomes from ICC patients (21), there is a high level of correlation (*r* = 0.690; *P* < 0.0001) between those with a high expression of genes associated with Wnt signaling and high expression of genes associated with AKT signaling (Fig. 4A; Supplementary Table S9). Therefore, we considered whether the activation of Wnt and PI3K signaling is a more universal process in ICC formation and sought to address whether these pathways are recurrently activated in ICC lacking *RAS* mutations, particularly given the majority of ICC patient cancers (79.07%) do not carry a mutation in either *Kras* or *Nras* (Supplementary Table S4).

To test this, we deleted *Trp53*, which is the most common mutation in our patient cohort, mutated in 19.13% of cases (Fig. 1C) and *Pten* specifically in biliary epithelial cells using a *Keratin19-Cre^ERT^* knock-in mouse line. Although *Pten* mutations were not found in our computational analysis of ICC, loss of *Pten* recapitulates the effects of PI3KCA mutations (which is mutated in 5.4% of patients in our cohort, Fig. 1C; ref. 40), resulting in the accumulation of PIP_3_ and AKT activation. Furthermore, previous studies have demonstrated that *Pten* loss potentiates ICC formation on the background of steatosis (fatty liver) and interacts with *Smad4*-loss to initiate liver cancer (41, 42). Concurrently, with *Trp53* and *Pten* deletion, recombined cells were labeled with tdTomato (from here on in this line is known as the KPPTom line, Fig. 4B). These animals are then challenged with chronic administration of Thioacetamide, a hepatotoxin that has been used to induce ICC in other models but is not itself mutagenic (43). Within 4 weeks of Thioacetamide treatment, mice develop moderate to well-differentiated cholangiocellular neoplasms around the portal tracts within the liver, which are lineage traced with tdTomato, confirming that they have arisen from the biliary epithelium (Fig. 4C). Using FUnGI imaging (44), we found that small, tdTomato-positive ICC microfoci (∼200 μm across) can be identified forming from the ducts by 4 weeks and in normal 2D histology appear as small clusters of lineage traced ducts, with an atypical luminal structure. These cancerous microfoci grow progressively over 8 weeks and occupy approximately 22% of the liver (Fig. 4D). In this model, the deletion of *Trp53* and *Pten* alone are insufficient to promote tumor initiation, instead the addition of a damaging agent (Thioacetamide) and the induction of ductular regeneration are essential for tumor initiation (Fig. 4D). Tumors from KPPTom mice are positive for dephosphorylated (active) β-catenin, which localizes to the cytoplasm and nucleus of cancer cells, as well as phosphorylated AKT^Ser647^ (Fig. 4E). As KPPTom mice show both Wnt and PI3K/AKT activity, we treated them with LGK974 (to inhibit Wnt signaling) and pictilisib (to inhibit Pi3K signaling) at 4 weeks following tumor induction (i.e., the start of Thioacetamide treatment), and a time point at which we know that there is ICC forming in the liver (Fig. 4F), to determine whether a model of well-differentiated ICC is susceptible to coinhibition of these pathways. Following treatment, the number of tdTomato-positive cancer cells was significantly reduced (by 68.3%) when compared with control vehicle treated animals (Fig. 4G and H). Indeed, when treated with LGK974 and pictilisib, only 33% of KPPTom mice developed ICC, whereas in the vehicle treated cohort, 80% of KPPTom mice contained cancerous lesions (Fig. 4I).

## Discussion

ICC is highly complex at the genetic (25) and cellular levels. Although a number of candidate mutations have been identified that can be pharmacologically targeted (19), these have not led to a broadly applicable treatment. Moreover, the presence of these mutations does not necessarily predict therapeutic responsiveness in patients with ICC and only a subset of these patients respond to targeted therapy (20). Consequently, there is a clinical necessity to identify the mechanisms that ICC uses to grow and define whether these processes can be used in patient stratification and targeted treatment. To define a targeted therapeutic approach that can be used in treating a specific cohort of patients with ICC, we need to understand whether the genetic complexity found in ICC ultimately translates to phenotypic diversity or whether ICC relies on a limited number of signaling cascades to grow in spite of this genetic complexity.

The identification of causative mutations in ICC is fraught with complications. Historically these studies relied heavily on identification of genes with recurrent consensus mutations in patients. This approach has been severely limited by the relatively small number of ICC samples available. Increasing sample size by pooling data from across published cohorts and combining this with a driver prediction pipeline that puts less weight on mutation recurrence within a population, but rather concentrates on the patterns and predicted effects of mutations (10) enabled us to identify an expanded set of candidate mutations in ICC that have the potential to act as oncogenes.

Although able to predict novel candidate oncogenic mutations, the approach described here is not able to infer which mutations act in epistasis to promote tumor formation. To overcome this limitation we developed an *in vivo*, highly multiplexed CRISPR-SpCas9 screening approach. Our strategy builds on previous work demonstrating that smaller gene-editing libraries can be used to define which tumor suppressors are important for ICC initiation and build on a substantial body of work identifying which oncogenes are essential for the formation of cancer cells in the liver (26), particularly KRas, which has previously been used to define the responsiveness to MEK inhibition in ICC (45). Rather than using whole genome screening, we biased our libraries to ensure that they targeted genes mutated in patients with ICC. Using this strategy has enabled us to define a range of putative tumor suppressors that cooperate with gain-of-function *Ras*-mutations to overcome RAS-induced senescence and initiate tumor formation. We validated that one of these, *Nf2* or Merlin, can act as an important factor in the initiation of RAS-driven ICC and we show increased penetrance and aggression of RAS-driven ICC when *Nf2* loss is combined with *Trp53* loss. The phenotypes of these *Nf2*-mutant tumors are highly sarcomatoid, the cancer cells have a more spindle-like morphology and are dispersed throughout the liver. Perhaps this is unsurprising given the role of NF2 in maintaining contact inhibition and restricting cell migration in the presence of Ras mutations, as NF2 has been implicated in the fine tuning of Ras signaling in Schwann cells (46). However, loss of *Nf2* has not been shown to be sufficient to cooperate with Ras and drive tumor formation independent of other accessory mutations and whereas loss of *Nf2* has been previously shown to promote the formation of mixed HCC and ICC when it is deleted from the liver during development (47), it was unclear whether *de novo* somatic *Nf2* mutations interact with other ICC-relevant mutations *in vivo*. In addition to Hippo signaling, NF2 has also been implicated in the regulation of a number of other signaling pathways (48). We found that Wnt and PI3K signaling are recurrently deregulated following *Nf2* loss in our mouse model, leading us to identify coinhibition of Wnt and PI3K as a potent therapeutic combination in reducing ICC growth. In cancer, Wnt signaling is classically activated by mutations in a core set of genes (49). In hepatocellular carcinoma for example, the Wnt signaling pathway is activated via gain-of-function mutations in β-catenin and is known to directly promote tumor progression in mice (50). No such classical activating mutations have been found in ICC genome sequencing (with the exception of rare RNF43 mutations (8)) despite a number of descriptions of ICC being a Wnt-high tumor (35). Our data indicates that other mutations can similarly potentiate Wnt signaling likely by enabling cancer cells to enter a state that is permissive to ligand reception. Critically, we show that Wnt signaling is part of a signaling network that also involves the activation of AKT, which in ICC can be activated through a number of mechanisms and is known to affect the stability of β-catenin by inhibitory phosphorylation of GSK3β. Furthermore, Wnt (porcupine) inhibitors and PI3K inhibitors are currently in clinical trials for other solid malignancies and our data supports recent findings in pancreatic cancer (51), that treating patients with Wnt and PI3K inhibitors provides an attractive therapeutic route to reduce tumor burden.

## Conclusions

Our data demonstrate the importance of understanding the function of rare mutations in ICC and show that these low-frequency mutations not only influence the outcome of more common driver mutations, but also can lead us to define applicable therapeutic strategies that can be used to develop personalized approaches that could be applied clinically to stratify patients and to treat ICC of divergent genotypes based on the signaling pathways that are deregulated in these cancers.

## Authors' Disclosures

E. Jarman reports grants from Leap Therapeutics, BASL, and CCF outside the submitted work. P. Tennant reports grants from Medical Research Council during the conduct of the study. T.J. Kendall reports personal fees from Incyte Corporation outside the submitted work. M.S. Taylor reports grants from Medical Research Council during the conduct of the study and grants from Wellcome Trust outside the submitted work. No disclosures were reported by the other authors.

## Supplementary Material

Supplementary Data

Supplementary Data

Supplementary Data

## References

[1] Banales JM , Marin JJG, Lamarca A, Rodrigues PM, Khan SA, Roberts LR, . Cholangiocarcinoma 2020: the next horizon in mechanisms and management. Nat Rev Gastroenterol Hepatol 2020;17:557–88. 32606456 10.1038/s41575-020-0310-z PMC7447603

[2] Valle JW , Kelley RK, Nervi B, Oh D-Y, Zhu AX. Biliary tract cancer. Lancet 2021;397:428–44. 33516341 10.1016/S0140-6736(21)00153-7

[3] Adeva J , Sangro B, Salati M, Edeline J, La Casta A, Bittoni A, . Medical treatment for cholangiocarcinoma. Liver Int 2019;39:123–42. 30892822 10.1111/liv.14100

[4] Petmitr S , Pinlaor S, Thousungnoen A, Karalak A, Migasena P. K-ras oncogene and p53 gene mutations in cholangiocarcinoma from Thai patients. Southeast Asian J Trop Med Public Health 1998;29:71–75. 9740272

[5] Hill MA , Alexander WB, Guo B, Kato Y, Patra K, O'Dell MR, . Kras and Tp53 mutations cause cholangiocyte- and hepatocyte-derived cholangiocarcinoma. Cancer Res 2018;78:4445–51. 29871934 10.1158/0008-5472.CAN-17-1123 PMC6097629

[6] Goeppert B , Folseraas T, Roessler S, Kloor M, Volckmar A-L, Endris V, . Genomic characterization of cholangiocarcinoma in primary sclerosing cholangitis reveals therapeutic opportunities. Hepatology 2020;72:1253–66. 31925805 10.1002/hep.31110

[7] Zou S , Li J, Zhou H, Frech C, Jiang X, Chu JSC, . Mutational landscape of intrahepatic cholangiocarcinoma. Nat Commun 2014;5:5696. 25526346 10.1038/ncomms6696

[8] Chan-On W , Nairismägi M-L, Ong CK, Lim WK, Dima S, Pairojkul C, . Exome sequencing identifies distinct mutational patterns in liver fluke-related and non-infection-related bile duct cancers. Nat Genet 2013;45:1474–8. 24185513 10.1038/ng.2806

[9] Jusakul A , Cutcutache I, Yong CH, Lim JQ, Huang MN, Padmanabhan N, . Whole-genome and epigenomic landscapes of etiologically distinct subtypes of cholangiocarcinoma. Cancer Discov 2017;7:1116–35. 28667006 10.1158/2159-8290.CD-17-0368 PMC5628134

[10] Gonzalez-Perez A , Perez-Llamas C, Deu-Pons J, Tamborero D, Schroeder MP, Jene-Sanz A, . IntOGen-mutations identifies cancer drivers across tumor types. Nat Methods 2013;10:1081–2. 24037244 10.1038/nmeth.2642 PMC5758042

[11] Sia D , Losic B, Moeini A, Cabellos L, Hao K, Revill K, . Massive parallel sequencing uncovers actionable FGFR2-PPHLN1 fusion and ARAF mutations in intrahepatic cholangiocarcinoma. Nat Commun 2015;6:6087. 25608663 10.1038/ncomms7087

[12] Shalem O , Sanjana NE, Hartenian E, Shi X, Scott DA, Mikkelson T, . Genome-scale CRISPR-Cas9 knockout screening in human cells. Science 2014;343:84–87. 24336571 10.1126/science.1247005 PMC4089965

[13] Read A , Gao S, Batchelor E, Luo J. Flexible CRISPR library construction using parallel oligonucleotide retrieval. Nucleic Acids Res 2017;45:e101. 28334828 10.1093/nar/gkx181 PMC5499874

[14] Ewels PA , Peltzer A, Fillinger S, Patel H, Alneberg J, Wilm A, . The nf-core framework for community-curated bioinformatics pipelines. Nat Biotechnol 2020;38:276–8. 32055031 10.1038/s41587-020-0439-x

[15] Thorvaldsdóttir H , Robinson JT, Mesirov JP. Integrative Genomics Viewer (IGV): high-performance genomics data visualization and exploration. Brief Bioinformatics 2013;14:178–92. 22517427 10.1093/bib/bbs017 PMC3603213

[16] Rios AC , Capaldo BD, Vaillant F, Pal B, van Ineveld R, Dawson CA, . Intraclonal plasticity in mammary tumors revealed through large-scale single-cell resolution 3D imaging. Cancer Cell 2019;35:618–32. 30930118 10.1016/j.ccell.2019.02.010

[17] Sriskandarajah P , De Haven Brandon A, MacLeod K, Carragher NO, Kirkin V, Kaiser M, . Combined targeting of MEK and the glucocorticoid receptor for the treatment of RAS-mutant multiple myeloma. BMC Cancer 2020;20:269. 32228485 10.1186/s12885-020-06735-2 PMC7106683

[18] Lowery MA , Burris HA, Janku F, Shroff RT, Cleary JM, Azad NS, . Safety and activity of ivosidenib in patients with IDH1-mutant advanced cholangiocarcinoma: a phase 1 study. Lancet Gastroenterol Hepatol 2019;4:711–20. 31300360 10.1016/S2468-1253(19)30189-X PMC7934945

[19] Javle MM , Borbath I, Clarke SJ, Hitre E, Louvet C, Mercade TM, . Infigratinib versus gemcitabine plus cisplatin multicenter, open-label, randomized, phase 3 study in patients with advanced cholangiocarcinoma with FGFR2 gene fusions/translocations: the PROOF trial. JCO 2019;37:TPS4155.

[20] Bekaii-Saab TS , Bridgewater J, Normanno N. Practical considerations in screening for genetic alterations in cholangiocarcinoma. Ann Oncol 2021;32:1111–26. 33932504 10.1016/j.annonc.2021.04.012

[21] Nakamura H , Arai Y, Totoki Y, Shirota T, Elzawahry A, Kato M, . Genomic spectra of biliary tract cancer. Nat Genet 2015;47:1003–10. 26258846 10.1038/ng.3375

[22] Farshidfar F , Zheng S, Gingras M-C, Newton Y, Shih J, Robertson AG, . Integrative genomic analysis of cholangiocarcinoma identifies distinct IDH-mutant molecular profiles. Cell Rep 2017;19:2878–80. 28658632 10.1016/j.celrep.2017.06.008 PMC6141445

[23] Wu G , Feng X, Stein L. A human functional protein interaction network and its application to cancer data analysis. Genome Biol 2010;11:R53. 20482850 10.1186/gb-2010-11-5-r53 PMC2898064

[24] Newman MEJ . Modularity and community structure in networks. Proc Natl Acad Sci USA 2006;103:8577–82. 16723398 10.1073/pnas.0601602103 PMC1482622

[25] Dong L-Q , Shi Y, Ma L-J, Yang L-X, Wang X-Y, Zhang S, . Spatial and temporal clonal evolution of intrahepatic cholangiocarcinoma. J Hepatol 2018;69:89–98. 29551704 10.1016/j.jhep.2018.02.029

[26] Weber J , Öllinger R, Friedrich M, Ehmer U, Barenboim M, Steiger K, . CRISPR/Cas9 somatic multiplex-mutagenesis for high-throughput functional cancer genomics in mice. Proc Natl Acad Sci USA 2015;112:13982–7. 26508638 10.1073/pnas.1512392112 PMC4653208

[27] Wang J , Dong M, Xu Z, Song X, Zhang S, Qiao Y, . Notch2 controls hepatocyte-derived cholangiocarcinoma formation in mice. Oncogene 2018;37:3229–42. 29545603 10.1038/s41388-018-0188-1 PMC6002343

[28] Fan B , Malato Y, Calvisi DF, Naqvi S, Razumilava N, Ribback S, . Cholangiocarcinomas can originate from hepatocytes in mice. J Clin Invest 2012;122:2911–5. 22797301 10.1172/JCI63212 PMC3408746

[29] Tennant PA , Foster RG, Dodd DO, Sou IF, McPhie F, Younger N, . Fluorescent *in vivo* editing reporter (FIVER): a novel multispectral reporter of *in vivo* genome editing. BioRxiv 2020.

[30] Kang T-W , Yevsa T, Woller N, Hoenicke L, Wuestefeld T, Dauch D, . Senescence surveillance of pre-malignant hepatocytes limits liver cancer development. Nature 2011;479:547–51. 22080947 10.1038/nature10599

[31] Morton JP , Timpson P, Karim SA, Ridgway RA, Athineos D, Doyle B, . Mutant p53 drives metastasis and overcomes growth arrest/senescence in pancreatic cancer. Proc Natl Acad Sci USA 2010;107:246–51. 20018721 10.1073/pnas.0908428107 PMC2806749

[32] Kim M , Kim S, Lee SH, Kim W, Sohn MJ, Kim HS, . Merlin inhibits Wnt/β-catenin signaling by blocking LRP6 phosphorylation. Cell Death Differ 2016;23:1638–47. 27285107 10.1038/cdd.2016.54 PMC5041192

[33] Malhotra S , Wood J, Mansy T, Singh R, Zaitoun A, Madhusudan S. Intrahepatic sarcomatoid cholangiocarcinoma. J Oncol 2010;2010:701476. 20454704 10.1155/2010/701476 PMC2862318

[34] Kim DK , Kim BR, Jeong JS, Baek YH. Analysis of intrahepatic sarcomatoid cholangiocarcinoma: experience from 11 cases within 17 years. World J Gastroenterol 2019;25:608–21. 30774275 10.3748/wjg.v25.i5.608 PMC6371010

[35] Boulter L , Guest RV, Kendall TJ, Wilson DH, Wojtacha D, Robson AJ, . WNT signaling drives cholangiocarcinoma growth and can be pharmacologically inhibited. J Clin Invest 2015;125:1269–85. 25689248 10.1172/JCI76452 PMC4362247

[36] Guichard C , Amaddeo G, Imbeaud S, Ladeiro Y, Pelletier L, Maad IB, . Integrated analysis of somatic mutations and focal copy-number changes identifies key genes and pathways in hepatocellular carcinoma. Nat Genet 2012;44:694–8. 22561517 10.1038/ng.2256 PMC3819251

[37] Sato K , Murai H, Ueda Y, Katsuda S. Intrahepatic sarcomatoid cholangiocarcinoma of round cell variant: a case report and immunohistochemical studies. Virchows Arch 2006;449:585–90. 17033799 10.1007/s00428-006-0291-5

[38] Mah KM , Houston DW, Weiner JA. The γ-Protocadherin-C3 isoform inhibits canonical Wnt signalling by binding to and stabilizing Axin1 at the membrane. Sci Rep 2016;6:31665. 27530555 10.1038/srep31665 PMC4987702

[39] O'Rourke CJ , Matter MS, Nepal C, Caetano-Oliveira R, Ton PT, Factor VM, . Identification of a pan-gamma-secretase inhibitor response signature for notch-driven cholangiocarcinoma. Hepatology 2020;71:196–213. 31211856 10.1002/hep.30816 PMC6918012

[40] Carnero A , Blanco-Aparicio C, Renner O, Link W, Leal JFM. The PTEN/PI3K/AKT signalling pathway in cancer, therapeutic implications. Curr Cancer Drug Targets 2008;8:187–98. 18473732 10.2174/156800908784293659

[41] Xu X , Kobayashi S, Qiao W, Li C, Xiao C, Radaeva S, . Induction of intrahepatic cholangiocellular carcinoma by liver-specific disruption of Smad4 and Pten in mice. J Clin Invest 2006;116:1843–52. 16767220 10.1172/JCI27282 PMC1474816

[42] Chen J , Debebe A, Zeng N, Kopp J, He L, Sander M, . Transformation of SOX9+ cells by Pten deletion synergizes with steatotic liver injury to drive development of hepatocellular and cholangiocarcinoma. Sci Rep 2021;11:11823. 34083580 10.1038/s41598-021-90958-1 PMC8175600

[43] Guest RV , Boulter L, Dwyer BJ, Kendall TJ, Man T-Y, Minnis-Lyons SE, . Notch3 drives development and progression of cholangiocarcinoma. Proc Natl Acad Sci USA 2016;113:12250–5. 27791012 10.1073/pnas.1600067113 PMC5086988

[44] Dawson CA , Pal B, Vaillant F, Gandolfo LC, Liu Z, Bleriot C, . Tissue-resident ductal macrophages survey the mammary epithelium and facilitate tissue remodelling. Nat Cell Biol 2020;22:546–58. 32341550 10.1038/s41556-020-0505-0

[45] Wang P , Song X, Utpatel K, Shang R, Yang YM, Xu M, . MEK inhibition suppresses K-Ras wild-type cholangiocarcinoma *in vitro* and *in vivo* via inhibiting cell proliferation and modulating tumor microenvironment. Cell Death Dis 2019;10:120. 30741922 10.1038/s41419-019-1389-4 PMC6370758

[46] Cui Y , Groth S, Troutman S, Carlstedt A, Sperka T, Riecken LB, . The NF2 tumor suppressor merlin interacts with Ras and RasGAP, which may modulate Ras signaling. Oncogene 2019;38:6370–81. 31312020 10.1038/s41388-019-0883-6 PMC6756068

[47] Benhamouche-Trouillet S , O'Loughlin E, Liu C-H, Polacheck W, Fitamant J, McKee M, . Proliferation-independent role of NF2 (merlin) in limiting biliary morphogenesis. Development 2018;145:dev162123. 29712669 10.1242/dev.162123 PMC10682933

[48] Attisano L , Wrana JL. Signal integration in TGF-β, WNT, and Hippo pathways. F1000Prime Rep 2013;5:17. 23755364 10.12703/P5-17 PMC3672943

[49] Monga SP . β-Catenin signaling and roles in liver homeostasis, injury, and tumorigenesis. Gastroenterology 2015;148:1294–310. 25747274 10.1053/j.gastro.2015.02.056 PMC4494085

[50] Qiao Y , Wang J, Karagoz E, Liang B, Song X, Shang R, . Axis inhibition protein 1 (Axin1) deletion-induced hepatocarcinogenesis requires intact β-catenin but not notch cascade in mice. Hepatology 2019;70:2003–17. 30737831 10.1002/hep.30556 PMC7206928

[51] Zhong Z , Sepramaniam S, Chew XH, Wood K, Lee MA, Madan B, . PORCN inhibition synergizes with PI3K/mTOR inhibition in Wnt-addicted cancers. Oncogene 2019;38:6662–77. 31391551 10.1038/s41388-019-0908-1 PMC10200719

