# Supplementary material for: In Vivo Modeling of Patient Genetic Heterogeneity Identifies New Ways to Target Cholangiocarcinoma
Source: Cancer Res. 2022 Jan 24;82(8):1548–59. doi: 10.1158/0008-5472.CAN-21-2556 (PMC9359731; doi:10.1158/0008-5472.CAN-21-2556)
Supplement: Supplementary Data [file can-21-2556_supplementary_data_suppsm.docx]

**Supplementary material:**

**Title:**  *In vivo* modeling of patient genetic heterogeneity identifies new ways to target cholangiocarcinoma.

**Authors:** Nicholas T. Younger, Mollie L. Wilson, Anabel Martinez Lyons, Edward J. Jarman, Alison M. Meynert, Graeme R Grimes, Konstantinos Gournopanos, Scott H. Waddell, Peter A. Tennant, David H. Wilson, Rachel V. Guest, Stephen J. Wigmore, Juan Carlos Acosta, Timothy J. Kendall, Martin S. Taylor, Duncan Sproul, Pleasantine Mill, Luke Boulter

**Supplementary Methods:**

**Identification and processing of genomic data from patient datasets:**

Alignment and pre-processing of publicly available data: Exome-seq FASTQ files from Chan-on et al. (1) were downloaded from the European Nucleotide Archive with accession PRJEB4445. Exome-seq FASTQ files from Sia et al. (2) were downloaded from the Gene Expression Omnibus Database with accession GSE63420. TCGA BAM files were downloaded from the Genomic Data Commons after receiving access to individual patient BAM files.

For the TCGA, Chan-On, and Sia datasets, mutations were called as follows. Input FASTQ files were aligned to the Hg19 reference genome using Burrows-Wheeler Aligner version 0.7.15(3). PCR and optical duplicates were marked using PicardMarkDuplicates version 2.5.0, base quality score recalibration was carried out using BaseRecalibrator and local indel realignment was performed with IndelRealigner (both from the Genome Analysis Toolkit(4) version 3.6 (GATK3)). Ensemble variant calling was performed with Mutect (1.1.5)(5), Mutect2 (2.1)(6), Freebayes (1.0.2.29)(7), Vardict-java (1.4.6)(8), and Varscan (2.4.2)(9) and all passing somatic variants which were identified by 2 or more algorithms were taken forward. The above analysis was batched and implemented within the Blue Collar Bioinformatics pipeline (bcb io-nextgen 1.0.0a0-af4730e). TCGA data were input as reads which were aligned to the Hg38 reference so, following mutation calling, variants were re-mapped using Picard LiftoverVCF (2.5.0) using the Hg38toHg19 chain file provided by UCSC. Variant annotation was performed with the Ensembl Variant Effect Predictor (v88) using the ‘--pick’, ‘--tab’, and ‘--symbol’ options.

Identification of candidate pathogenic variants using IntOgen: IntOgen(10) was run on each published/TCGA cohort individually, and then all combined. Significance thresholds for OncoDriveFM and OncoDriveCLUST were q=0.05, and q=0.1 for MutSigCV as per the default recommendations. The minimum frequency of occurrence for a gene to be considered for analysis was n=2 for OncoDriveFM and n=5 for OncoDriveCLUST. Following candidate identification with IntOgen, functional Interaction Inference Cytoscape(11) (3.7.1) with the ReactomeFIVis(12) app was used to build a network of known and inferred functional interactions between the set of predicted drivers so as to explore the relationship between them in a cellular context. This network was clustered into modules by connection density and each module was annotated with pathway enrichments.

Evolutionary dependency (co-occurrence and mutual exclusivity) was scored using SELECT(13) (version 1.6) with default parameters. The weighted mutual information (wMI) p-value was used for colour coding significance and false discovery rate (FDR) <0.1 used as multi-testing corrected threshold of significance(14). For the aggregate analysis of genes (“cosmic” and “other” groups), individual tumours were binary encoded as 1 if they contained a putative driver mutation in any gene of the corresponding gene list, and 0 otherwise.

**CRISPR/Cas9-editing validation and Structural Variant calling:** DNA sequences were extracted from FASTQ files from exome-sequencing of tumours arising from the RAS^G12^-library screens and were aligned to the FVB mouse reference genome; subsequently, indels within 50bp upstream or downstream of sgRNA target sites were called. To determine if indels were likely due to SpCas9 activity or were spurious mutations, the interval of each indel was observed on the FVB genome using Integrative Genomics Viewer(15), and overlain with sgRNA library binding sites. Indels with start or end sites out-with sgRNA targets were removed, although this was only the case for 5 indels across all samples. To determine if editing had induced structural rearrangements, structural variants (SVs) were called using Delly2 (v0.7.9) (16)as follows: all reads which had a minimum mapping quality of 20 were used to call germline and somatic SVs, SVs with a PASS flag for somatic status, had a minimum variant allele fraction of 0.15, had zero read support in the normal, and had precisely mapped breakpoints were then visually analysed on IGV to determine if they overlapped with sgRNA target sites.

**Data curation and deposition:** All RNA and Exome sequencing data pertaining to this manuscript is deposited on the NCBI Gene Expression Omnibus (GEO) as accession number GSE190770.

**References:**

1. Chan-On W, Nairismägi M-L, Ong CK, Lim WK, Dima S, Pairojkul C, et al. Exome sequencing identifies distinct mutational patterns in liver fluke-related and non-infection-related bile duct cancers. Nat Genet. 2013 Dec;45(12):1474–1478.

2. Sia D, Losic B, Moeini A, Cabellos L, Hao K, Revill K, et al. Massive parallel sequencing uncovers actionable FGFR2-PPHLN1 fusion and ARAF mutations in intrahepatic cholangiocarcinoma. Nat Commun. 2015 Jan 22;6:6087.

3. Li H, Durbin R. Fast and accurate short read alignment with Burrows-Wheeler transform. Bioinformatics. 2009 Jul 15;25(14):1754–1760.

4. DePristo MA, Banks E, Poplin R, Garimella KV, Maguire JR, Hartl C, et al. A framework for variation discovery and genotyping using next-generation DNA sequencing data. Nat Genet. 2011 May;43(5):491–498.

5. Cibulskis K, Lawrence MS, Carter SL, Sivachenko A, Jaffe D, Sougnez C, et al. Sensitive detection of somatic point mutations in impure and heterogeneous cancer samples. Nat Biotechnol. 2013 Mar;31(3):213–219.

6. Benjamin DI, Sato T, Cibulskis K, Getz G, Stewart C, Lichtenstein L. Calling Somatic SNVs and Indels with Mutect2. BioRxiv. 2019 Dec 2;

7. Garrison E, Marth G. Haplotype-based variant detection from short-read sequencing. arXiv. 2012 Jul 17;

8. Lai Z, Markovets A, Ahdesmaki M, Chapman B, Hofmann O, McEwen R, et al. VarDict: a novel and versatile variant caller for next-generation sequencing in cancer research. Nucleic Acids Res. 2016 Jun 20;44(11):e108.

9. Koboldt DC, Chen K, Wylie T, Larson DE, McLellan MD, Mardis ER, et al. VarScan: variant detection in massively parallel sequencing of individual and pooled samples. Bioinformatics. 2009 Sep 1;25(17):2283–2285.

10. Gonzalez-Perez A, Perez-Llamas C, Deu-Pons J, Tamborero D, Schroeder MP, Jene-Sanz A, et al. IntOGen-mutations identifies cancer drivers across tumor types. Nat Methods. 2013 Nov;10(11):1081–1082.

11. Shannon P, Markiel A, Ozier O, Baliga NS, Wang JT, Ramage D, et al. Cytoscape: a software environment for integrated models of biomolecular interaction networks. Genome Res. 2003 Nov;13(11):2498–2504.

12. Wu G, Feng X, Stein L. A human functional protein interaction network and its application to cancer data analysis. Genome Biol. 2010 May 19;11(5):R53.

13. Mina M, Raynaud F, Tavernari D, Battistello E, Sungalee S, Saghafinia S, et al. Conditional selection of genomic alterations dictates cancer evolution and oncogenic dependencies. Cancer Cell. 2017 Aug 14;32(2):155–168.e6.

14. Sanchez-Vega F, Mina M, Armenia J, Chatila WK, Luna A, La KC, et al. Oncogenic signaling pathways in the cancer genome atlas. Cell. 2018 Apr 5;173(2):321–337.e10.

15. Thorvaldsdóttir H, Robinson JT, Mesirov JP. Integrative Genomics Viewer (IGV): high-performance genomics data visualization and exploration. Brief Bioinformatics. 2013 Mar;14(2):178–192.

16. Rausch T, Zichner T, Schlattl A, Stütz AM, Benes V, Korbel JO. DELLY: structural variant discovery by integrated paired-end and split-read analysis. Bioinformatics. 2012 Sep 15;28(18):i333–i339.

| Oligo Schematic for library pool synthesis | | | | |
| --- | --- | --- | --- | --- |
|  | | | | |
| Forward Primer binding arm | 5' Esp3I cut site | 5'->3' sequence | 5' Esp3I cut site | Reverse Primer binding arm |
| GCACAGTTTCACGATCGCTTTAGC | CGTCTCACACCG | N(20) | GTTTTGAGACG | CTGTCGTGGAATCGCTAAAGCAAG |
|  |  |  |  |  |
| Primers for isolating gRNAs from library pool | | | | |
| Forward | GCACAGTTTCACGATCGCTTTAGC | Reverse | CTTGCTTTAGCGATTCCACGACAG | |
|  |  |  |  |  |

| gRNAs included within pool | | |  |
| --- | --- | --- | --- |
| Gene Name | Target ID | gRNA sequence 5'-3' |  |
| Abl1 | Abl1_1 | CCCTTCATACCGCAGCGAGA |  |
| Abl1 | Abl1_2 | AAGGGAGGGTGTACCACTAC |  |
| Abl1 | Abl1_3 | CGAGAGCCGCTTCAACACTC |  |
| Ablim1 | Ablim1_1 | GTCATAGATCGCCTTGACCT |  |
| Ablim1 | Ablim1_2 | GAAAGTATCTATTCTAGACC |  |
| Ablim1 | Ablim1_3 | CTTACAGTCGGGATGCCACA |  |
| Acvr1b | Acvr1b_1 | GTGTCTACCATAACCGCCAG |  |
| Acvr1b | Acvr1b_2 | GTCTACGACCTCTCCACGTC |  |
| Acvr1b | Acvr1b_3 | AGGGCCGGTTCGGGGAAGTA |  |
| Akap9 | Akap9_1 | GATGCCGTCTCGCTGTTTAA |  |
| Akap9 | Akap9_2 | GCCGCCATTAAACAGCGAGA |  |
| Akap9 | Akap9_3 | GCGCACCTGCTTCAGCCCTT |  |
| Apbb1 | Apbb1_1 | CGAGACGGATTCCGATCTAC |  |
| Apbb1 | Apbb1_2 | GGAATCCGTCTCGAAAGCGT |  |
| Apbb1 | Apbb1_3 | GAAGGACAATGTCGCCTCCT |  |
| Aqp7 | Aqp7_1 | ACTCCCCAGGCATTCGTGAC |  |
| Aqp7 | Aqp7_2 | GGCATCCTTGTTACCGTCCT |  |
| Aqp7 | Aqp7_3 | CATTCGGCCTAGTGCACAAT |  |
| Arhgap8 | Arhgap8_1 | GGCCCTCACCGCACTACTTC |  |
| Arhgap8 | Arhgap8_2 | TCCTCCAGGGGATGACCGCC |  |
| Arhgap8 | Arhgap8_3 | TATAGTCGTTCTCCACATGC |  |
| Arid1a | Arid1a_1 | GCCCTGCTGGCCATACGCAC |  |
| Arid1a | Arid1a_2 | GGTCCCTGTTGTTGCGAGTA |  |
| Arid1a | Arid1a_3 | TAACCCATACTCGCAACAAC |  |
| Arid2 | Arid2_1 | TACCAGCAACACAGCGTGTC |  |
| Arid2 | Arid2_2 | ATAGCGAAGTCCACTTCATT |  |
| Arid2 | Arid2_3 | CACTTTACTGCTCGCTAATG |  |
| Atf7ip | Atf7ip_1 | CCGTACGTACTTCTCACAGG |  |
| Atf7ip | Atf7ip_2 | TCCAAAACGTTTGGTTAACC |  |
| Atf7ip | Atf7ip_3 | GGCAGTGCTCACCGAGCTGC |  |
| Atp2b1 | Atp2b1_1 | TTACGTGCATTATCCCCTTC |  |
| Atp2b1 | Atp2b1_2 | AATTGCAGCCATAGTATCAT |  |
| Atp2b1 | Atp2b1_3 | CCTGCTCAATTCGACTCTGC |  |
| Atp7a | Atp7a_1 | AACACGGTATTGGTTAAGAC |  |
| Atp7a | Atp7a_2 | CAAGGTGTTCAGCGCATTAA |  |
| Atp7a | Atp7a_3 | GCAGCCGAAGTACCTCAAAT |  |
| Bap1 | Bap1_1 | TCAAATGGATCGAAGAGCGC |  |
| Bap1 | Bap1_2 | CCGCCGCAAGGTTTCTACGT |  |
| Bap1 | Bap1_3 | GCCCACGCTGAGCCGAATGA |  |
| Bcor | Bcor_1 | GATGAGGCCGAATCCAACGA |  |
| Bcor | Bcor_2 | CTTACGGGCTGCATACGAGC |  |
| Bcor | Bcor_3 | CGACGCTTCAAAAGCCAGGC |  |
| Braf | Braf_1 | TCATAGGTACCCGCAAGATG |  |
| Braf | Braf_2 | GAGGCCCTATTGGACAAATT |  |
| Braf | Braf_3 | GTTGTTCCGTGATGCATCTG |  |
| Brca2 | Brca2_1 | TAGGACCGATAAGCCTCAAT |  |
| Brca2 | Brca2_2 | AAAGCTCCTCAAACCAATTG |  |
| Brca2 | Brca2_3 | GGACTAGCAACATCTACCAC |  |
| Cacna1c | Cacna1c_1 | CAACCGCCACACGGCCGCCC |  |
| Cacna1c | Cacna1c_2 | CTCCTCGCAGGTTCCAACTA |  |
| Cacna1c | Cacna1c_3 | GTTGGAGTCGTCTTCCGGAA |  |
| Camkk1 | Camkk1_1 | ACAGGCCGTCCTCCCCCCAG |  |
| Camkk1 | Camkk1_2 | TCTCCTGATACACACGCTCC |  |
| Camkk1 | Camkk1_3 | TCTGTCTTCACTTTCGTTGT |  |
| Cc2d2a | Cc2d2a_1 | ACTCACTCCGGGGCCCCCGC |  |
| Cc2d2a | Cc2d2a_2 | AGGCAGAAAGCGCATTGTTG |  |
| Cc2d2a | Cc2d2a_3 | CAACAATGCGCTTTCTGCCT |  |
| Ccdc66 | Ccdc66_1 | TGAATACTGCTTATCCGTAC |  |
| Ccdc66 | Ccdc66_2 | CACTACCGGTACTTGTCCAG |  |
| Ccdc66 | Ccdc66_3 | TGCTCATGTTCACTCGTGAC |  |
| Cdk18 | Cdk18_1 | GCCCTGCACTGCTATTCGAG |  |
| Cdk18 | Cdk18_2 | GAACCAGCGCCGATTCTCCA |  |
| Cdk18 | Cdk18_3 | TCCCAGTCAGATATCGGCTT |  |
| Cdkn2a | Cdkn2a_1 | GTGCGATATTTGCGTTCCGC |  |
| Cdkn2a | Cdkn2a_2 | CCCAACGCCCCGAACTCTTT |  |
| Cdkn2a | Cdkn2a_3 | GGGGTACGACCGAAAGAGTT |  |
| Chd4 | Chd4_1 | GCTTCGGATAAATCCTCGTC |  |
| Chd4 | Chd4_2 | TCGGACCCTCACCAACTACA |  |
| Chd4 | Chd4_3 | TCCTGACTTACCTAACAAAT |  |
| Cped1 | Cped1_1 | TAGTCGACACATGTGCTCCC |  |
| Cped1 | Cped1_2 | TGGTCTAGGTCCGGAGCTGC |  |
| Cped1 | Cped1_3 | GTCGACTAAGCCTACATCAA |  |
| Cpt1a | Cpt1a_1 | GACTATGCGCTACTCGCTGA |  |
| Cpt1a | Cpt1a_2 | CACTCACGATGTTCTTCGTC |  |
| Cpt1a | Cpt1a_3 | GGGTTACTCACGTGGTGTCT |  |
| Dbndd2 | Dbndd2_1 | CATAGGTAGCATCTCGTCTA |  |
| Dbndd2 | Dbndd2_2 | GTTTATTGATCTTGCGGATC |  |
| Dbndd2 | Dbndd2_3 | GATGTGGTCCTGTCAGACGT |  |
| Dchs1 | Dchs1_1 | TAGGTAGGGCTATCAAATCG |  |
| Dchs1 | Dchs1_2 | TGTGTGTACCACCCGGACCC |  |
| Dchs1 | Dchs1_3 | TAGAGCGCGCAGTGTATAGA |  |
| Ece2 | Ece2_1 | GAACGTCGCGCTGCACGAGT |  |
| Ece2 | Ece2_2 | CTCGTGCAGCGCGACGTTCA |  |
| Ece2 | Ece2_3 | GACCATGGATGTTCGAGCAC |  |
| Ehhadh | Ehhadh_1 | AGCTGCGTTCCTCTTGCACC |  |
| Ehhadh | Ehhadh_2 | CTATCGGATTGCCAATGCAA |  |
| Ehhadh | Ehhadh_3 | GCCATCCAAGGCGTGGCTCT |  |
| Elf3 | Elf3_1 | CGACTTCTCCCGCTGCGACA |  |
| Elf3 | Elf3_2 | CTAGCGAGCGGCCCCAGTTC |  |
| Elf3 | Elf3_3 | CAAAGGGGCCCGAGTCGCCT |  |
| Epha2 | Epha2_1 | CAACGTGGTATCCGGCGACC |  |
| Epha2 | Epha2_2 | TTCGCTGTCGAAGCACGCAA |  |
| Epha2 | Epha2_3 | CTGCTGACCGTGATCTCGTC |  |
| Erbb2 | Erbb2_1 | CTTACAGGCCCGGGAACGAT |  |
| Erbb2 | Erbb2_2 | GCTAGACAACCGAGACCCTT |  |
| Erbb2 | Erbb2_3 | ACATGGACACCAATCGTTCC |  |
| Erbb3 | Erbb3_1 | CCCCTTGCAGACTTCGTGAC |  |
| Erbb3 | Erbb3_2 | CGGGGAACCCAGGTCTACGA |  |
| Erbb3 | Erbb3_3 | GCCCTTACCTAACCTCCGAG |  |
| Esrra | Esrra_1 | TGTACTTCTGCCGTCCGCCG |  |
| Esrra | Esrra_2 | GTACGTCCTGCTGAAAGCTC |  |
| Esrra | Esrra_3 | TGCGACACCAGAGCGTTCAC |  |
| Etv6 | Etv6_1 | GCATGGCGTGCTCTTCCGGT |  |
| Etv6 | Etv6_2 | GTGAACATGAAGCGGAGTCG |  |
| Etv6 | Etv6_3 | CTGACAGGGGGTACGTTTCC |  |
| Fam136a | Fam136a_1 | GCGCCGCTACCTGCATCTTC |  |
| Fam136a | Fam136a_2 | CGAGCGCTGCCATGCGCCTC |  |
| Fam136a | Fam136a_3 | CATGGCAGCGCTCGATGCAT |  |
| Fam174b | Fam174b_1 | CCACGATCACCGTCGCCTTC |  |
| Fam174b | Fam174b_2 | TCGCAGAGCCGAGTCCGCTT |  |
| Fam174b | Fam174b_3 | GCCATTTCCACACGTTCCGC |  |
| Fh1 | Fh1_1 | AATTGGGCGAACTCACACGC |  |
| Fh1 | Fh1_2 | CGTGTAGAGTTCGACACCTT |  |
| Fh1 | Fh1_3 | AAAATCCAAAGAGTTTGCGC |  |
| Fn1 | Fn1_1 | CCAGGTCTCCCCCACGACGT |  |
| Fn1 | Fn1_2 | GACCTACCTAGGCAACGCCC |  |
| Fn1 | Fn1_3 | CCTACAAGATTGGCGACAAG |  |
| Fzd2 | Fzd2_1 | ATCGACGGCGACCTGCTGAG |  |
| Fzd2 | Fzd2_2 | GGCGCGGTGGTGAGTAGCGC |  |
| Fzd2 | Fzd2_3 | TGCGAGCATTTCCCGCGTCA |  |
| Gpr162 | Gpr162_1 | AGTCCAACGGCTCGGCTATC |  |
| Gpr162 | Gpr162_2 | TCTCCGGATAGCCGAGCCGT |  |
| Gpr162 | Gpr162_3 | GTGTGTGGCGATCATGTCCG |  |
| Gstp1 | Gstp1_1 | GTCAGCCAGCAGCATTCGCA |  |
| Gstp1 | Gstp1_2 | ATTGTCTACTTCCCAGTTCG |  |
| Gstp1 | Gstp1_3 | TCACATAGTTGGTGTAGATG |  |
| Heatr3 | Heatr3_1 | CACTACCAGACTTAGCCCGT |  |
| Heatr3 | Heatr3_2 | GCCGGCGCACAGCATCCCGA |  |
| Heatr3 | Heatr3_3 | ATTGACCTGGCTGTCTCCGT |  |
| Helz | Helz_1 | GCAACGAGTAATGTCCTGTC |  |
| Helz | Helz_2 | CTTCTACGTGACATCCAGAC |  |
| Helz | Helz_3 | GCTGATGAAGATTGTAGGCA |  |
| Hrct1 | Hrct1_1 | GAAACCGAGTCCGACAAGCC |  |
| Hrct1 | Hrct1_2 | GTGGTGGTGAACGCCCACAC |  |
| Hrct1 | Hrct1_3 | CGCCGGCCATGGAAGAGTCG |  |
| Idh1 | Idh1_1 | TGGGCCTGTAAGAATTACGA |  |
| Idh1 | Idh1_2 | GGCCCAAGCTATGAAGTCCG |  |
| Idh1 | Idh1_3 | AGTCTTCAATTGACTTATCC |  |
| Idh2 | Idh2_1 | ATGTTCCGGATCGTTCCGTT |  |
| Idh2 | Idh2_2 | AGAGCCCTAACGGAACGATC |  |
| Idh2 | Idh2_3 | TCGAGCTGGCACGTTCAAGT |  |
| Il1r1 | Il1r1_1 | AGTCCCGGTCCGCTGATATG |  |
| Il1r1 | Il1r1_2 | GCCGTATGTCCTATACGTTC |  |
| Il1r1 | Il1r1_3 | TTGCTTCCCCCGGAACGTAT |  |
| Itpr2 | Itpr2_1 | GGACATCGTGTCCCTGTACG |  |
| Itpr2 | Itpr2_2 | TGACCGAGCCCTCCGCGTAC |  |
| Itpr2 | Itpr2_3 | GTTCCCCTGTTTCGCCTGCT |  |
| Itpr3 | Itpr3_1 | AGCATCCAGCGTCACCCGCA |  |
| Itpr3 | Itpr3_2 | GCTGGATGCTACGGGCAATG |  |
| Itpr3 | Itpr3_3 | GGTCTTACCTCGGAACTTCT |  |
| Kras | Kras_1 | TGAGTATGACCCTACGATAG |  |
| Kras | Kras_2 | AGCAGCGTTACCTCTATCGT |  |
| Kras | Kras_3 | TAGAACAGTAGACACGAAAC |  |
| Mme | Mme_1 | TGCTCGACTGATTCAGAATA |  |
| Mme | Mme_2 | GGCCAGTAGCATCAGATAAC |  |
| Mme | Mme_3 | TCCCAGTTATCTGATGCTAC |  |
| Msr1 | Msr1_1 | GCGTTCCGTGTCTATAAGGT |  |
| Msr1 | Msr1_2 | TGAACGTGCGTCAAATTTCA |  |
| Msr1 | Msr1_3 | TTCCTTGATTTCGTCAGTCC |  |
| Mtch2 | Mtch2_1 | CTTTGACCGAGTTATCAAAG |  |
| Mtch2 | Mtch2_2 | GGCACCTACCATAGCAAAAG |  |
| Mtch2 | Mtch2_3 | AAAATATTTCGTCCTATTGT |  |
| Ncor1 | Ncor1_1 | GGATATGAACAGTTTCACTC |  |
| Ncor1 | Ncor1_2 | AATGTATTAGGCCTCAAGAA |  |
| Ncor1 | Ncor1_3 | TATGCCTTACCTGCTGGTGT |  |
| Nf2 | Nf2_1 | GATCCGCACCGTGAATGTCT |  |
| Nf2 | Nf2_2 | GAAGCTCATGCGAGAAGCGA |  |
| Nf2 | Nf2_3 | CTTGGCGTCATATGCTGTCC |  |
| Notch2 | Notch2_1 | GTCGTCGATATTCCGCTCAC |  |
| Notch2 | Notch2_2 | GCCTCCGTTGACGCAGGGCG |  |
| Notch2 | Notch2_3 | TACGAGTGCACCTGCCAAGT |  |
| Nprl3 | Nprl3_1 | AGCGGCGCTCCTCATGCTGC |  |
| Nprl3 | Nprl3_2 | CTGCCAGTACCTCACTCGAG |  |
| Nprl3 | Nprl3_3 | AAGGTTCTCGGATGTTATTC |  |
| Nras | Nras_1 | CTAGCATACCTGTCGGGTCT |  |
| Nras | Nras_2 | GACCTCAGCCAAGACCCGAC |  |
| Nras | Nras_3 | TTGCAGATATTAACCTCTAC |  |
| Pbrm1 | Pbrm1_1 | CAAACTCATTTCTTGTTCGA |  |
| Pbrm1 | Pbrm1_2 | GTTGTAGCCACAAATCCATC |  |
| Pbrm1 | Pbrm1_3 | ACATCACTTACCACTTTGGA |  |
| Phf20l1 | Phf20l1_1 | CACGATCCAGCTGGGTCGAT |  |
| Phf20l1 | Phf20l1_2 | GATATGATGAGTGGATTTAC |  |
| Phf20l1 | Phf20l1_3 | TGTTGTCCTATCGACCCAGC |  |
| Pik3ca | Pik3ca_1 | GCGCACTATTTATGACCCAG |  |
| Pik3ca | Pik3ca_2 | TCACCATGCCGTCATACTCC |  |
| Pik3ca | Pik3ca_3 | CAGAAGTCCAAGACTTTCGA |  |
| Pik3r1 | Pik3r1_1 | AGATGCGTCTCGTACCAAAA |  |
| Pik3r1 | Pik3r1_2 | GGAGTACACCCGTACTTCCC |  |
| Pik3r1 | Pik3r1_3 | GTGAATTACCTGCTGGTATT |  |
| Plch2 | Plch2_1 | TAGCCCGTCGCCAGCGTACC |  |
| Plch2 | Plch2_2 | CCGTAGTCCCCCAAGCCAAA |  |
| Plch2 | Plch2_3 | GATATTGGTCCCTGGTACGC |  |
| Plk2 | Plk2_1 | CAGAAGTCCGATACTACCTC |  |
| Plk2 | Plk2_2 | AAAACTGCACGACATGCTTA |  |
| Plk2 | Plk2_3 | ACGAACAAGAAATCTTGCAC |  |
| Plxnb2 | Plxnb2_1 | CTCAGATGGCCGGATCCTTA |  |
| Plxnb2 | Plxnb2_2 | TACTCTGCAGAAGTGCCGTC |  |
| Plxnb2 | Plxnb2_3 | GGAGTCACGACACTGAGCGC |  |
| Rasa1 | Rasa1_1 | ACACGCCTTCTATCTTCTAC |  |
| Rasa1 | Rasa1_2 | CTATAGCAGAAGAACGCCTC |  |
| Rasa1 | Rasa1_3 | CGTGACTGTAATAACCTATT |  |
| Rb1 | Rb1_1 | AGAAATCGATACCAGTACCA |  |
| Rb1 | Rb1_2 | TGACATAGCATTATCAACCT |  |
| Rb1 | Rb1_3 | TTGGGAGAAAGTTTCATCCG |  |
| Rbmx | Rbmx_1 | CCTTAAGCCCCTGTGTCCCG |  |
| Rbmx | Rbmx_2 | CTCTTGACTTATTCGTTTCT |  |
| Rbmx | Rbmx_3 | CGAGAAACGAATAAGTCAAG |  |
| Rev1 | Rev1_1 | ACGGCGTCTGCAATATTTAG |  |
| Rev1 | Rev1_2 | TGAAATGAAGTTGCAGTCCG |  |
| Rev1 | Rev1_3 | TTGGTCGTAGGGCGGGTACA |  |
| Rexo4 | Rexo4_1 | TGATACTTACTCAGTGGGCG |  |
| Rexo4 | Rexo4_2 | CCCTAGCTTGACAAAAGCCT |  |
| Rexo4 | Rexo4_3 | GTGTCGATCGTGAACCAGTA |  |
| Rnf31 | Rnf31_1 | CATACAACCGTAGTACATCC |  |
| Rnf31 | Rnf31_2 | AGGGTGGCCGGGATGTACTA |  |
| Rnf31 | Rnf31_3 | TAACCCCGTCTTTCGCAGCA |  |
| Rnf43 | Rnf43_1 | GAGACGCTTACCCCGGCGGG |  |
| Rnf43 | Rnf43_2 | CAGGGGCGAGGAGCTCGTCG |  |
| Rnf43 | Rnf43_3 | TTTCCACAGGCCCGAATGGC |  |
| Rps29 | Rps29_1 | CTCACCAAGAGCGGGAACCC |  |
| Rps29 | Rps29_2 | GAAGGACATAGGCTTCATTA |  |
| Rps29 | Rps29_3 | TGGGTCACCAGCAGCTCTAC |  |
| Sdk1 | Sdk1_1 | AGAACTCACGTGCTACGCTC |  |
| Sdk1 | Sdk1_2 | TCCTGTTCCGCACCACGCAG |  |
| Sdk1 | Sdk1_3 | CATCACTCGCTGTTGTAGCA |  |
| Setd2 | Setd2_1 | GATCTCTTTCGGACCGACAT |  |
| Setd2 | Setd2_2 | GTCGGTCCGAAAGAGATCGA |  |
| Setd2 | Setd2_3 | ACTGCATTCGCTTAATATCC |  |
| Sf3b1 | Sf3b1_1 | GTCCTCCAAAGATTGCCGAT |  |
| Sf3b1 | Sf3b1_2 | GCCTGGATATCATGCCCCCG |  |
| Sf3b1 | Sf3b1_3 | TATATCATTAAGCAACGCCA |  |
| Smad4 | Smad4_1 | GCCAAGTAATCGCGCATCAA |  |
| Smad4 | Smad4_2 | TCCGTTGATGCGCGATTACT |  |
| Smad4 | Smad4_3 | ACAACCCGCTCATAGTGATA |  |
| Smu1 | Smu1_1 | TCCAGATGAATATAGCGCTC |  |
| Smu1 | Smu1_2 | ATTGATAGAGCTTCGTGAAT |  |
| Smu1 | Smu1_3 | CTTGCCTCGAAACAAATCGA |  |
| Snrpn | Snrpn_1 | GGAACTCCACCTCCACCTGT |  |
| Snrpn | Snrpn_2 | TGGGGAATAGGTACACCTGC |  |
| Snrpn | Snrpn_3 | TTCACAGGTCATGACCCCAC |  |
| Stk11 | Stk11_1 | GCGCCCTACGTATATGGTGA |  |
| Stk11 | Stk11_2 | TGTACAGCACGTCCACAAGC |  |
| Stk11 | Stk11_3 | ATTCCAGGCCGTCAATCAGC |  |
| Tbc1d8b | Tbc1d8b_1 | CTAGGGACTAATCGCTGAAG |  |
| Tbc1d8b | Tbc1d8b_2 | GTCTGGTGTAGGATGCGAAA |  |
| Tbc1d8b | Tbc1d8b_3 | CCGTCCTCTCCAGTAACTGC |  |
| Tgfbr1 | Tgfbr1_1 | ATCTATTCAAGTAATCGAAA |  |
| Tgfbr1 | Tgfbr1_2 | CTTCTAGAGAAGAGCGTTCA |  |
| Tgfbr1 | Tgfbr1_3 | AGTGATGGATCCTCTTCATT |  |
| Thbs1 | Thbs1_1 | AAGGGGCCCCGGTCGCCGAC |  |
| Thbs1 | Thbs1_2 | CCCTTCACCAGTCGGCGACC |  |
| Thbs1 | Thbs1_3 | GCTATCCGCACCAACTACAT |  |
| Tmtc1 | Tmtc1_1 | GTAGAATGACATTGACCGCG |  |
| Tmtc1 | Tmtc1_2 | TTATACTCACCGCCTCAGTG |  |
| Tmtc1 | Tmtc1_3 | CCATGTCGCCGAGAGCTATG |  |
| Tmtc4 | Tmtc4_1 | GGCCTTTACCGGTGTACTGT |  |
| Tmtc4 | Tmtc4_2 | AGGATTAACTACTACCTGTC |  |
| Tmtc4 | Tmtc4_3 | CCCCAAGGGGCGTGTCTGAC |  |
| Trp53 | Trp53_1 | AGTGAAGCCCTCCGAGTGTC |  |
| Trp53 | Trp53_2 | AACAGATCGTCCATGCAGTG |  |
| Trp53 | Trp53_3 | TGAGGGCTTACCATCACCAT |  |
| Ttc8 | Ttc8_1 | CGATCTATGCACGCAGATGC |  |
| Ttc8 | Ttc8_2 | CGACCTATCACTAGCTCATC |  |
| Ttc8 | Ttc8_3 | CGGCCAGGTACCTGATCATA |  |
| Wdr62 | Wdr62_1 | ATGACATGGTCCTCAATGTT |  |
| Wdr62 | Wdr62_2 | GTCACTATGTACTTCCCATC |  |
| Wdr62 | Wdr62_3 | ATGTGCTTACCCTGCTAAGT |  |
| Zfp317 | Zfp317_1 | CCATAAATAGGTTATCAGGT |  |
| Zfp317 | Zfp317_2 | TTATAGCAATCTAAGTTCAC |  |
| Zfp317 | Zfp317_3 | GAACTACATCTAAAGTCAAA |  |
| Zfp36l2 | Zfp36l2_1 | CGATATCGACTTCTTGTGCA |  |
| Zfp36l2 | Zfp36l2_2 | CGCGTGCGCCAAACGCTCGC |  |
| Zfp36l2 | Zfp36l2_3 | GCCGCCGCTCGTCCGCGTTG |  |

**Supplementary methods table 1: Primer and gRNA sequences used in this study to generate gRNA libraries.**

| sgRNA | Mouse GeCKOv2 Library ID | 5'->3' Sequence |
| --- | --- | --- |
| SG0007 (NON-TARGETING) | MGLibA_66412 | ATTGTTCGACCGTCTACGGG |
| Trp53_1 | MGLibA_56033 | AGTGAAGCCCTCCGAGTGTC |
| Trp53_2 | MGLibA_56034 | AACAGATCGTCCATGCAGTG |
| Trp53_3 | MGLibA_56035 | TGAGGGCTTACCATCACCAT |
| Nf2_1 | MGLibA_33838 | GATCCGCACCGTGAATGTCT |
| Nf2_2 | MGLibA_33839 | GAAGCTCATGCGAGAAGCGA |
| Nf2_3 | MGLibA_33840 | CTTGGCGTCATATGCTGTCC |

**Supplementary methods table 2: gRNA sequences to target *Trp53* and *Nf2* or non-targeting sequence.**

| **Antigen** | **Manufacturer** | **Catalogue number** | **Usage** | **Methods** |
| --- | --- | --- | --- | --- |
| *Primary Antibodies* |  |  |  |  |
| active β-catenin | Cell Signalling | 19807 | Immunohistochemistry 1/200 | 4m Tris-EDTA + Tween20 pH9 pressure cooker |
| pAKTser647 | Cell Signalling | 4060 | Immunohistochemistry 1/25 | 20m Citrate buffer pH6, boiling waterbath |
| tdTomato | Abcam | ab62341 | Immunohistochemistry 1/100 | 10m Sodium Citrate microwave |
| Keratin-19 (Troma-III) | DSHB | Troma-III | Immunohistochemsitry 1/200 | 4m Tris-EDTA + Tween20 pH9 pressure cooker |
| GFP | Abcam | ab290 | Immunohistochemsitry 1/500 | 10m Tris-EDTA + Tween20 pH9 microwave |
| panCytokeratin | Dako | Z0622 | Immunohistochemsitry 1/200 | 5m Sodium Citrate followed by 5m ProteinaseK RT |
| *Secondary Antibodies* |  |  |  |  |
| Anti-Rabbit Biotin | Vector | BA-1000 | Immunohistochemistry 1/500 |  |
| Anti-Rat Biotin | Vector | BA-9400 | Immunohistochemistry 1/500 |  |
| Anti-Rabbit Alexa594 | Invitrogen | A-11012 | Immunohistochemistry 1/200 |  |
|  |  |  |  |  |

**Supplementary methods table 3: Antibodies used in this study.**

| **Target protein** | **Species** |
| --- | --- |
| 4E-BP1 P Ser65 | rabbit |
| Akt | rabbit |
| Akt P Ser473 | rabbit |
| AMPK alpha | rabbit |
| AMPK alpha P Thr172 | rabbit |
| Antibody | Type |
| ATM/ATR Substrate P Ser/Thr | rabbit |
| Aurora A/B/C P Thr288/Thr232/Thr198 | rabbit |
| Bad P Ser112 | rabbit |
| Bcl-2 | rabbit |
| Bcl-x | rabbit |
| beta-Catenin | rabbit |
| beta-Catenin P Ser33,Ser37,Thr41 | rabbit |
| beta-Tubulin | rabbit |
| Bid | rabbit |
| Bim P Ser69 | rabbit |
| c-Abl | rabbit |
| c-Abl P Y245 | rabbit |
| c-Abl P Y412 (247C7) | rabbit |
| Caspase 3 | rabbit |
| CDK1 (cdc2) | rabbit |
| Chk1 P Ser345 | rabbit |
| Cleaved Notch 1 (val1744) | rabbit |
| c-Myc | rabbit |
| c-Myc P Thr58,Ser62 | rabbit |
| CrkL | mouseIgG1 |
| CrkL P Tyr207 | rabbit |
| Cyclin D1 | mouseIgG2a |
| Cyclin D1 P Thr286 | rabbit |
| E-Cadherin | rabbit |
| EGFR P Tyr1173 | rabbit |
| EGFR P Y1068 | rabbit |
| EGFR P Y992 | rabbit |
| EphA2 (D4A2) | rabbit |
| EphA2 P Ser897 | rabbit |
| ErbB-1/EGFR | rabbit |
| ErbB-2/Her2/EGFR P Tyr1248/Tyr1173 | rabbit |
| Ezrin P T567/Radixin T564/Moesin T588 | rabbit |
| FLT3 P Tyr591 P Tyr591 | rabbit |
| FOXO1 (C29H4) | rabbit |
| FOXO3a (75D8) | rabbit |
| FRA1 (R20) | rabbit |
| Gab1 | rabbit |
| Grb2 | rabbit |
| Grb2 P Y237 | rabbit |
| GSK-3-alpha/beta P Ser21/Ser9 | rabbit |
| GSK-3-beta | rabbit |
| Histone H2A.X P Ser139 | mouseIgG1 |
| HSP27 (HSPB1) | mouseIgG1 |
| HSP27 (HSPB1) P Ser78 | rabbit |
| IGF-1R beta | rabbit |
| IGF-1R beta P Tyr1162,Tyr1163 | rabbit |
| IkB-alpha | rabbit |
| IKK alpha/beta P Ser176/Ser177 | rabbit |
| ILK1 (4G9) | rabbit |
| Integrin Beta 1 [EP1041Y] | rabbit |
| IRS-1 | rabbit |
| IRS-1 P S636/639 | rabbit |
| JAK1 | rabbit |
| JAK1 P Tyr1022,Thr1023 | rabbit |
| Ki-67 (Annexin II, p36) | mouseIgG1 |
| LKB1 | rabbit |
| MEK 1/2 P Ser217/221 | rabbit |
| MEK1/2 | rabbit |
| MEK1/2 P Ser217/221 | rabbit |
| MEK6 [EP558Y] | rabbit |
| Met | rabbit |
| Met P Tyr1234 | rabbit |
| Met P Tyr1349 | rabbit |
| MMP21 [EP1277Y] | rabbit |
| MNK1 (MKNK) P Thr197,Thr202 | rabbit |
| MNK1 (MKNK) P Thr197,Thr202 | rabbit |
| MSK1 P Ser376 | rabbit |
| mTOR (7C10) | rabbit |
| mTOR P Ser2448 | rabbit |
| p21 CIP/WAF1 | mouseIgG2a |
| p21 CIP/WAF1 p Thr145 | rabbit |
| p44/42 MAPK (ERK1/2) | rabbit |
| p44/42 MAPK (ERK1/2) P Thr202/Thr185,Tyr204/Tyr187 | rabbit |
| p53 | rabbit |
| p53 P Ser15 | rabbit |
| PARP | rabbit |
| PI3 Kinase p110-alpha | rabbit |
| PKC (pan) P Ser660 (beta-2) | rabbit |
| PKC-alpha | mouseIgG2b |
| PLC-gamma1 | rabbit |
| PLC-gamma1 P Tyr783 | rabbit |
| Profilin (C56B8) | rabbit |
| Prohibitin | rabbit |
| PTEN | rabbit |
| PTEN P Ser380,Thr382,Thr383 | rabbit |
| Puma | rabbit |
| PYK2 [EP206Y] | rabbit |
| PYK2 P Y402 | rabbit |
| Raf P Ser259 | rabbit |
| Rap1 | rabbit |
| Ras | mouseIgG1 |
| Rb | rabbit |
| Rb P Ser807,Ser811 | rabbit |
| Rb P Ser780 | rabbit |
| Rb P Ser780 | rabbit |
| RhoA (67BC) | rabbit |
| Rock1 (C8F7) | rabbit |
| SAPK/JNK (JNK2) | rabbit |
| SHP2 P Tyr542 | rabbit |
| Slug (C19G7 | rabbit |
| Smad1/5 P Ser463/Ser465 | rabbit |
| Smad2/3 P Ser465/Ser423,Ser467/Ser425 | rabbit |
| Src | rabbit |
| Src (family) P Tyr416 | rabbit |
| Stat1 P Ser727 | rabbit |
| Stat3 P Ser727 | rabbit |
| Stat3 P Tyr705 | mouseIgG1 |
| Stat5 | rabbit |
| Stat5 P Tyr694 | rabbit |
| Tau | rabbit |
| Tau Phospho/non Phos ser 305 | rabbit |
| TGF beta (56E4) | rabbit |
| VEGFR P Tyr1175 | rabbit |
| XIAP | rabbit |
| YAP1 [EP1674Y] | rabbit |

**Supplementary methods table 4: List of targets included in the RPPA screen.**

**
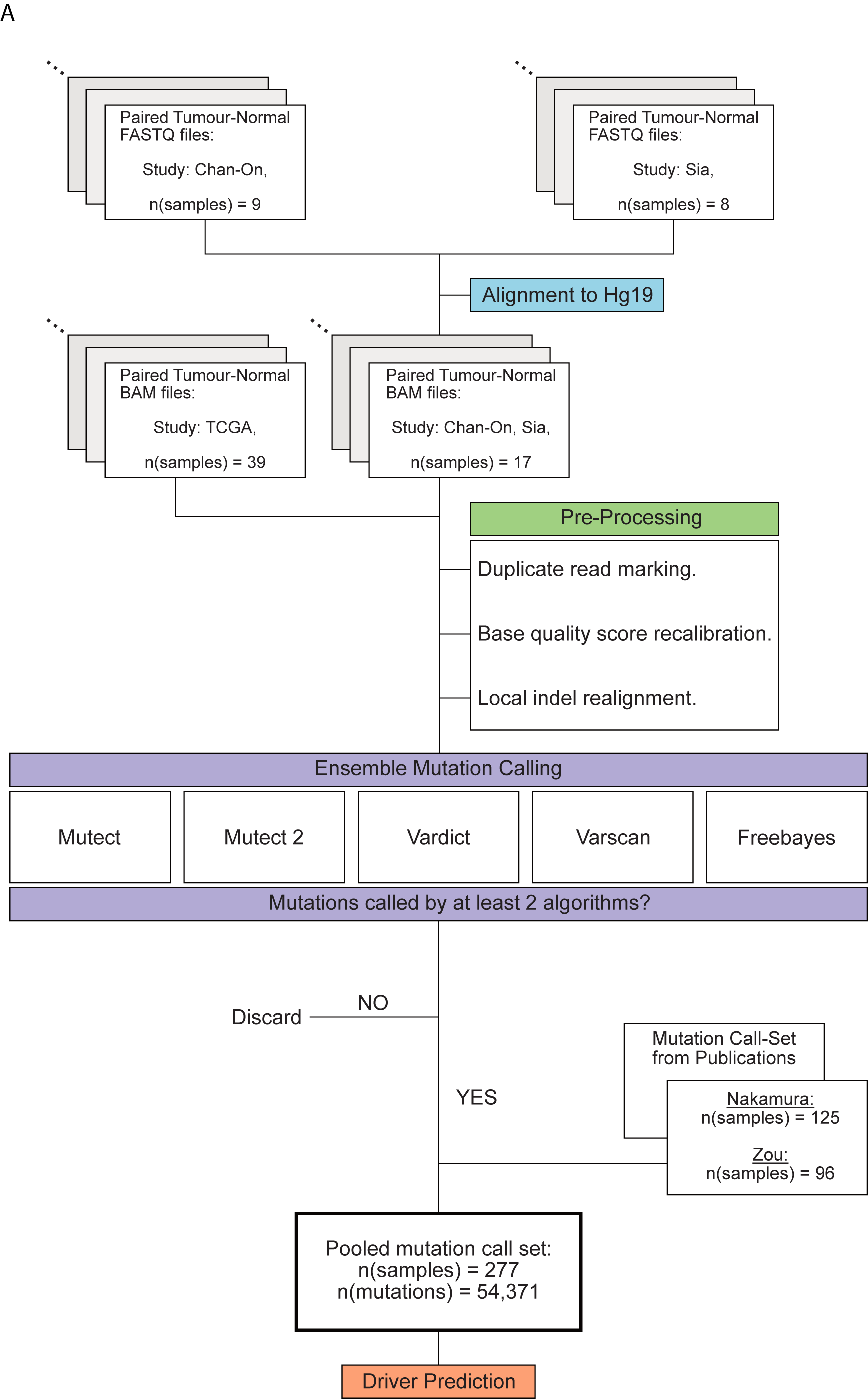
**

**Supplementary Figure 1 – Sequencing analysis pipeline:** Schematic representation of the data processing and data analysis pipeline applied to this study.


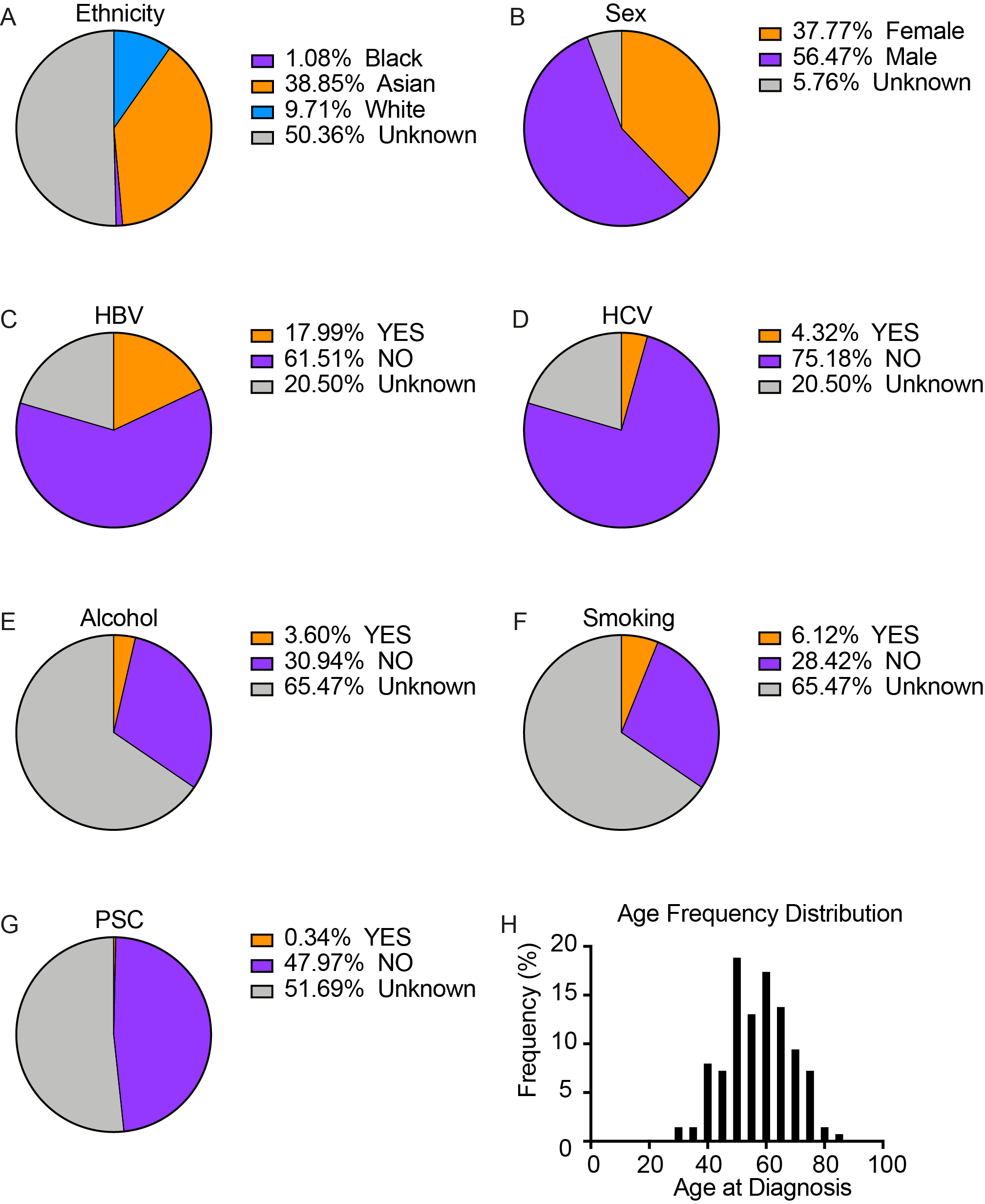


**Supplementary Figure 2 – Cohort Demographics:** **A.** Ethnic distribution of participants in this study (for those where the data is known). **B-G.** The proportion of each clinical attribute in the combined cohort. **H.** Distribution of ages of patients in the pooled cohort, the average is centred around 57 years.

**
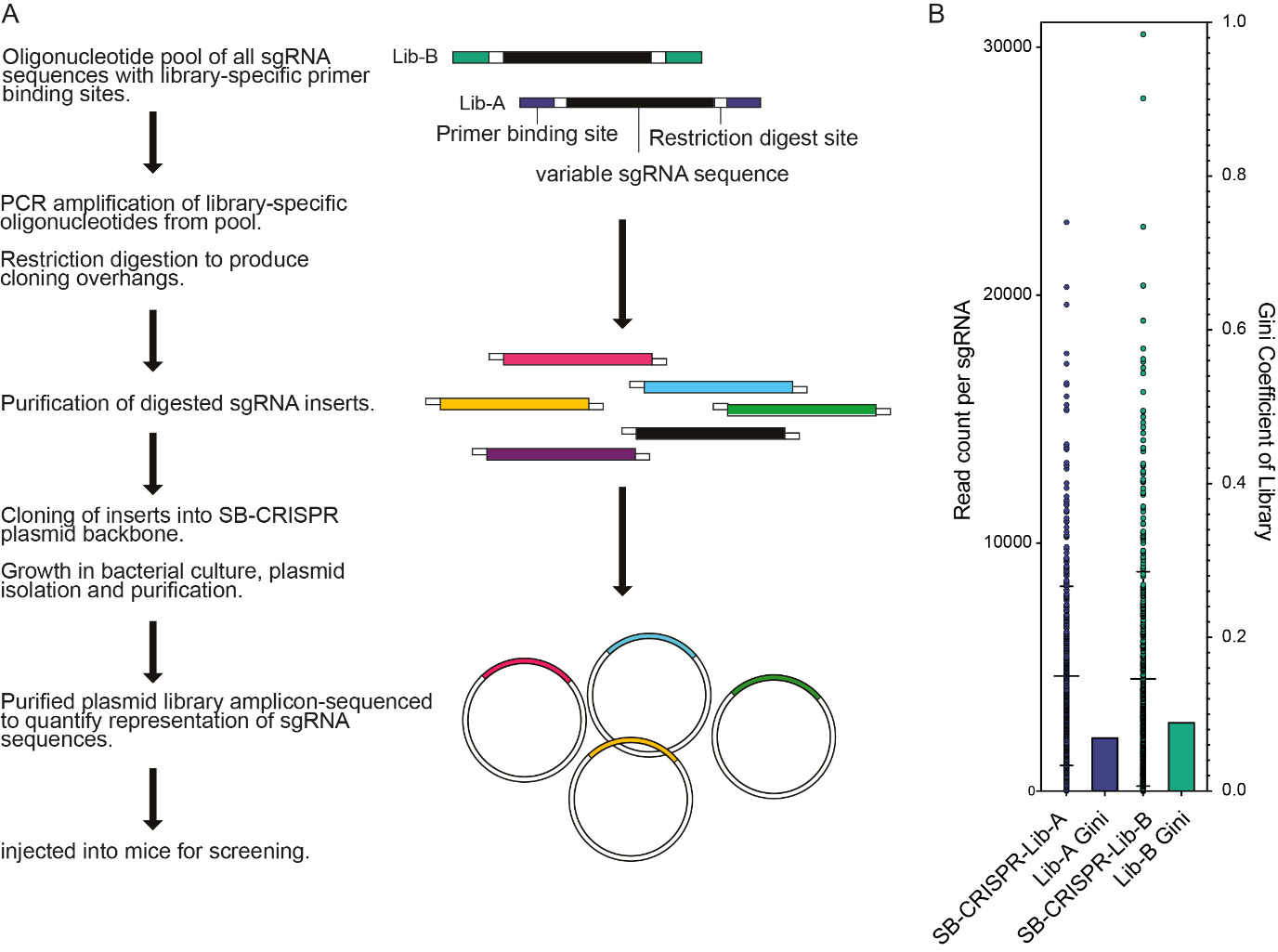
**

**Supplementary Figure 3 – Library generation and representation.** **A.** schematic of how the multiplexed ICC^Lib^ library is made; libraries are amplified from a pool and cloned into the SB-CRISPR backbone. **B.** MiSeq amplicon sequencing showed a low Gini index indicative of equal sgRNA sequence representation.


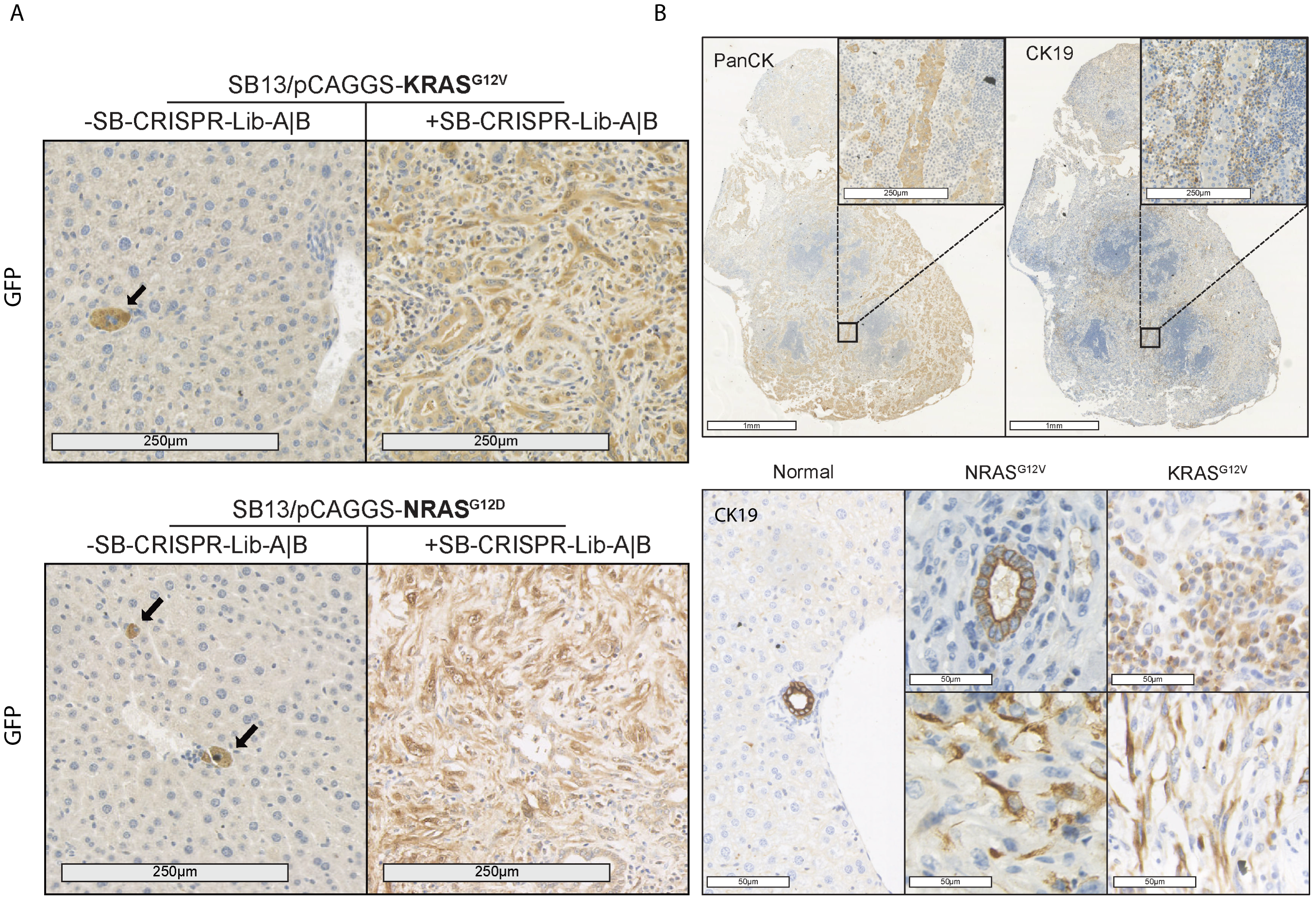


**Supplementary Figure 4 – Screened tumours are poorly differentiated adenocarcinomas with glandular features: A.** Immunohistochemistry for GFP (NRAS^G12V^ and KRAS^G12D^) expressing cells in mouse livers containing either a control vector (left panels) or SB-CRISPR Libraries (right panels). **B.** Immunohistochemistry of screened tumours for the epithelial marker pan-Cytokeratin (panCK), upper panels and the biliary lineage marker Cytokeratin-19 (CK19), lower panels in KRAS^G12D^ expressing tumours.


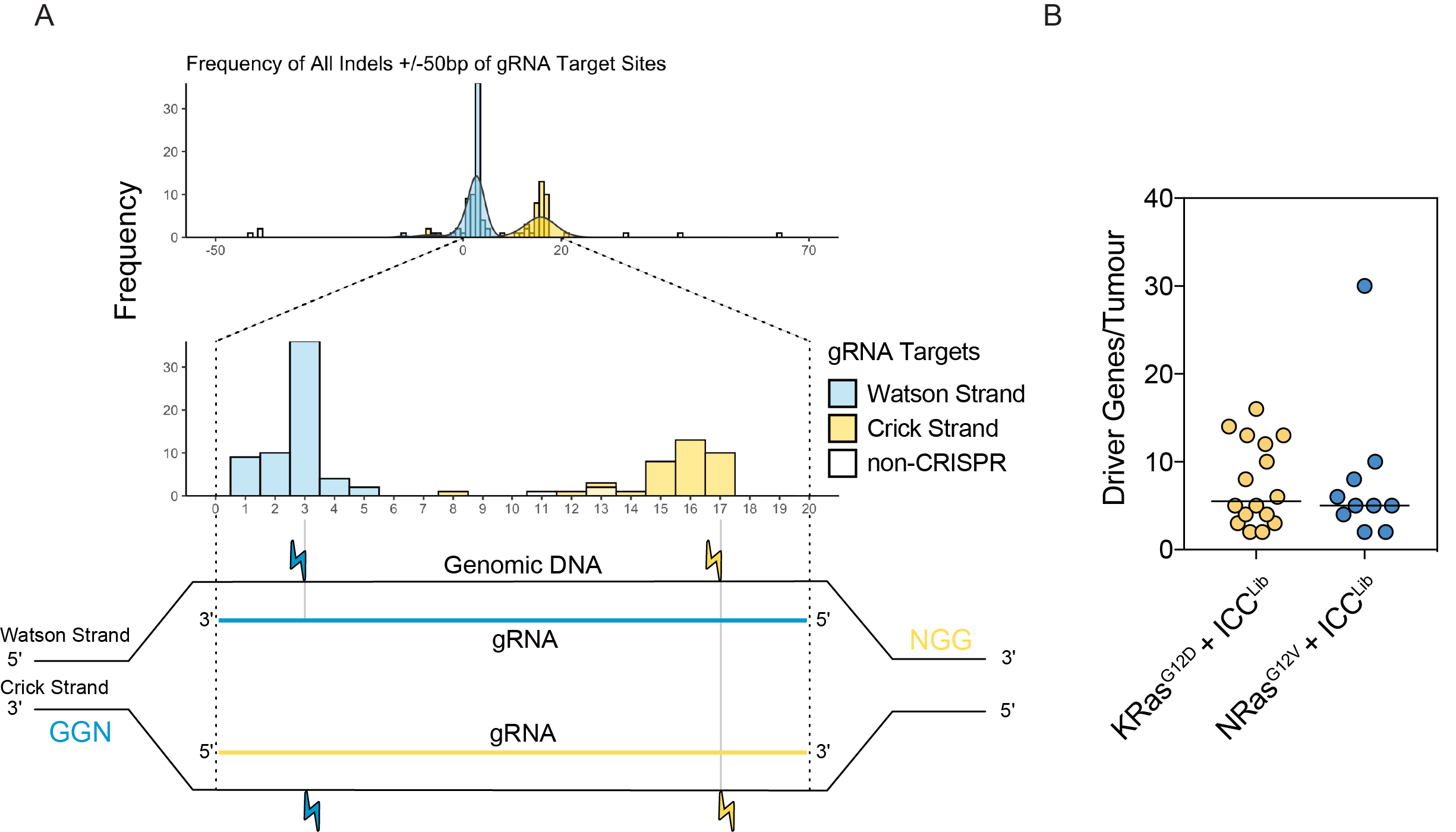


**Supplementary Figure 5 – Outcome of CRISPR-SpCas9 editing events:** **A.** indels within 50bp of sgRNA spacer target sites localise 2-4bp proximal to PAM sequences indicative of CRISPR/Cas9 mediated editing. **B.** The number of driver genes per tumourin in NRAS^G12V^ and KRAS^G12D^ oncogene screens when co injected with the ICC^Lib^.

**
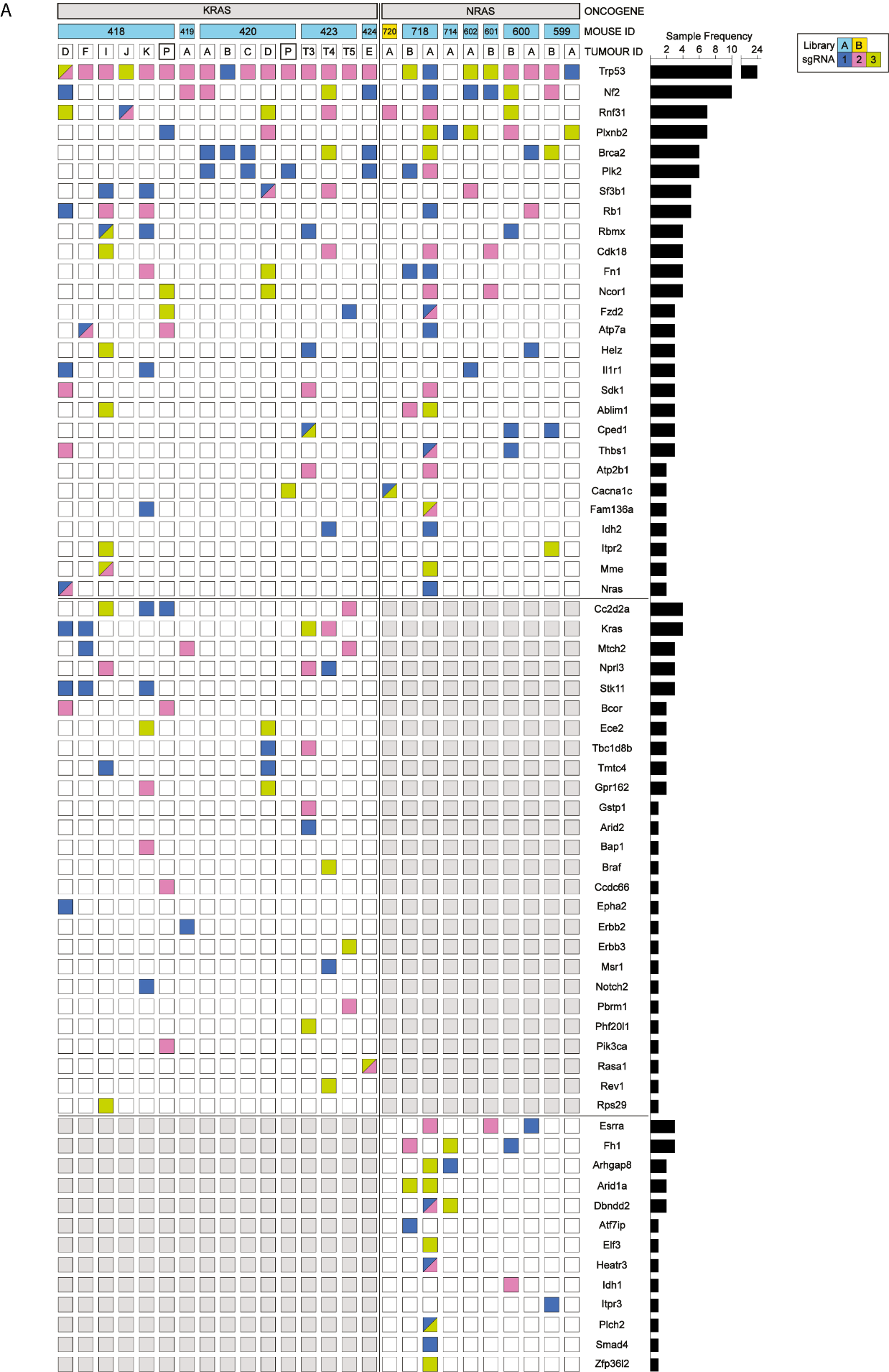
**

**Supplementary Figure 6 – Analysis of CRISPR-induced edits per tumour in exome-sequenced cancer:**  Tabulated results showing which genes are mutated in each of 26 tumours generated through the co-injection of either oncogenic NRAS^G12V^ or KRAS^G12D^ along with gRNA libraries targeting mutant ICC genes.


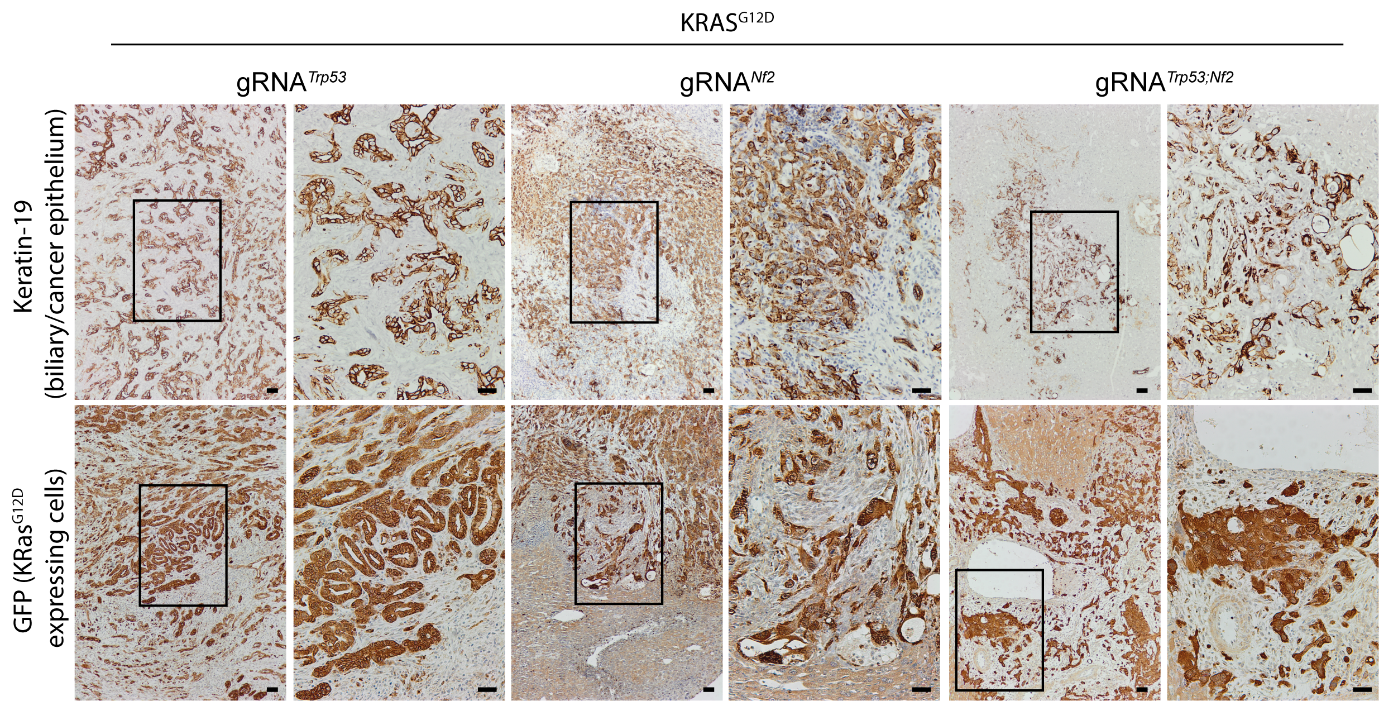


**Supplementary Figure 7 – Tumours arising following hydrodynamic injection have an ICC identity.** Upper panels show immunohistochemistry for the biliary marker Keratin-19. Lower panels show staining for GFP, denoting KRAS^G12D^-expressing cells. Boxes denote magnified regions in right hand panels. Scale bar = 100μm.

**
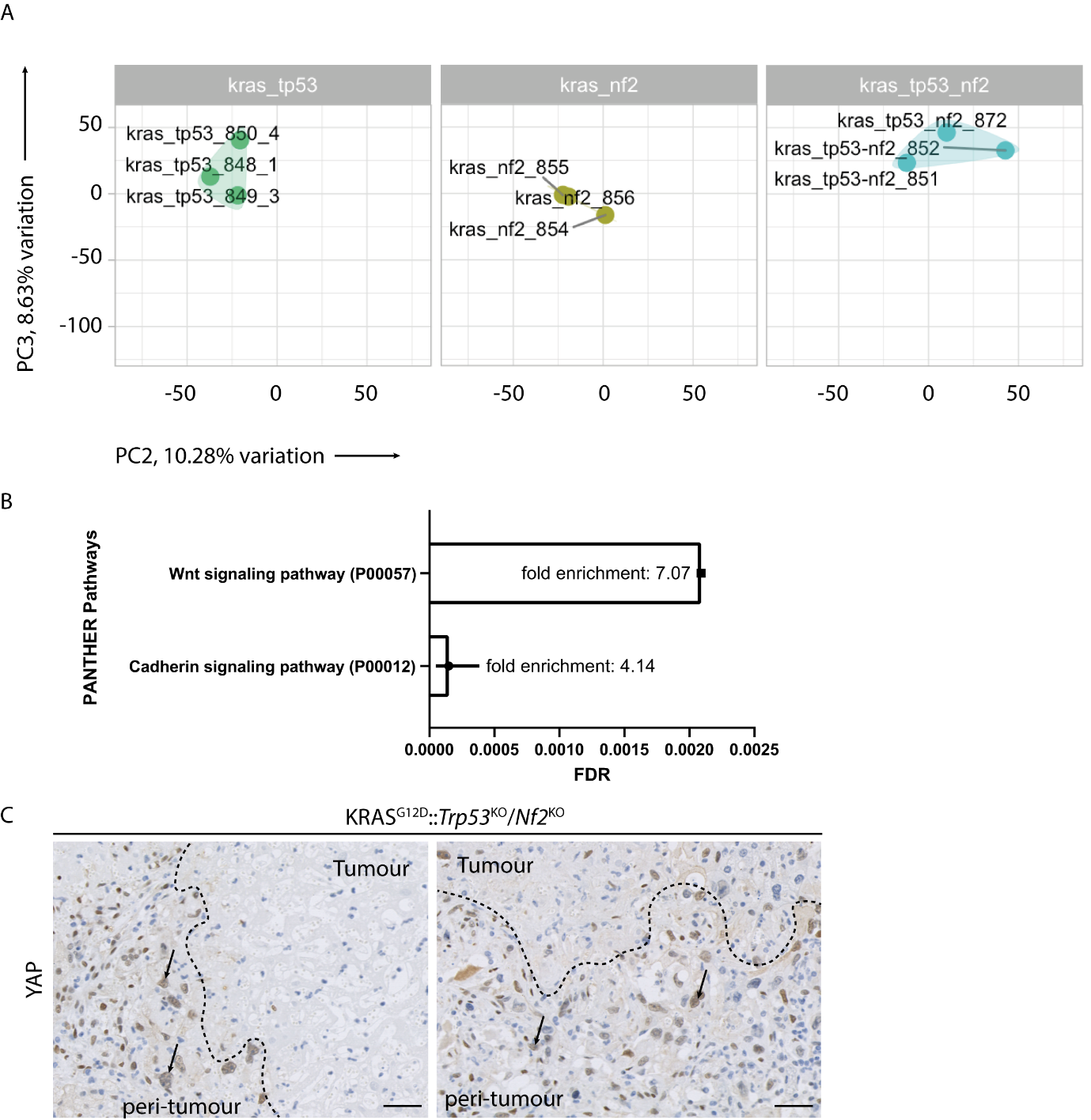
**

**Supplementary Figure 8 – Clustering of RNAseq data and PANTHER analysis:** **A.** Principal Component analysis of whole transcriptomes from KRAS^G12D^;*Trp53*^KO^, KRAS^G12D^;*Nf2*^KO^, KRAS^G12D^;*Trp53*^KO^;*Nf2*^KO^ tumours. **B.** Output of GO term analysis of significantly up and down regulated transcripts that are shared between groups when KRAS^G12D^;*Trp53*^KO^ is compared to KRAS^G12D^;*Trp53*^KO^/*Nf2*^KO ­^and when KRAS^G12D^;*Nf2*^KO^ is compared to KRAS^G12D^;*Trp53*^KO^;*Nf2*^KO^. **C.** Immunohistochemistry for dephosphorylated YAP in *Nf2*-deleted cancers. Black arrows denote positive nuclei, dotted line denotes the boundary between tumour and non-tumour tissue (labelled peri-tumour). Scale bar = 50μm.


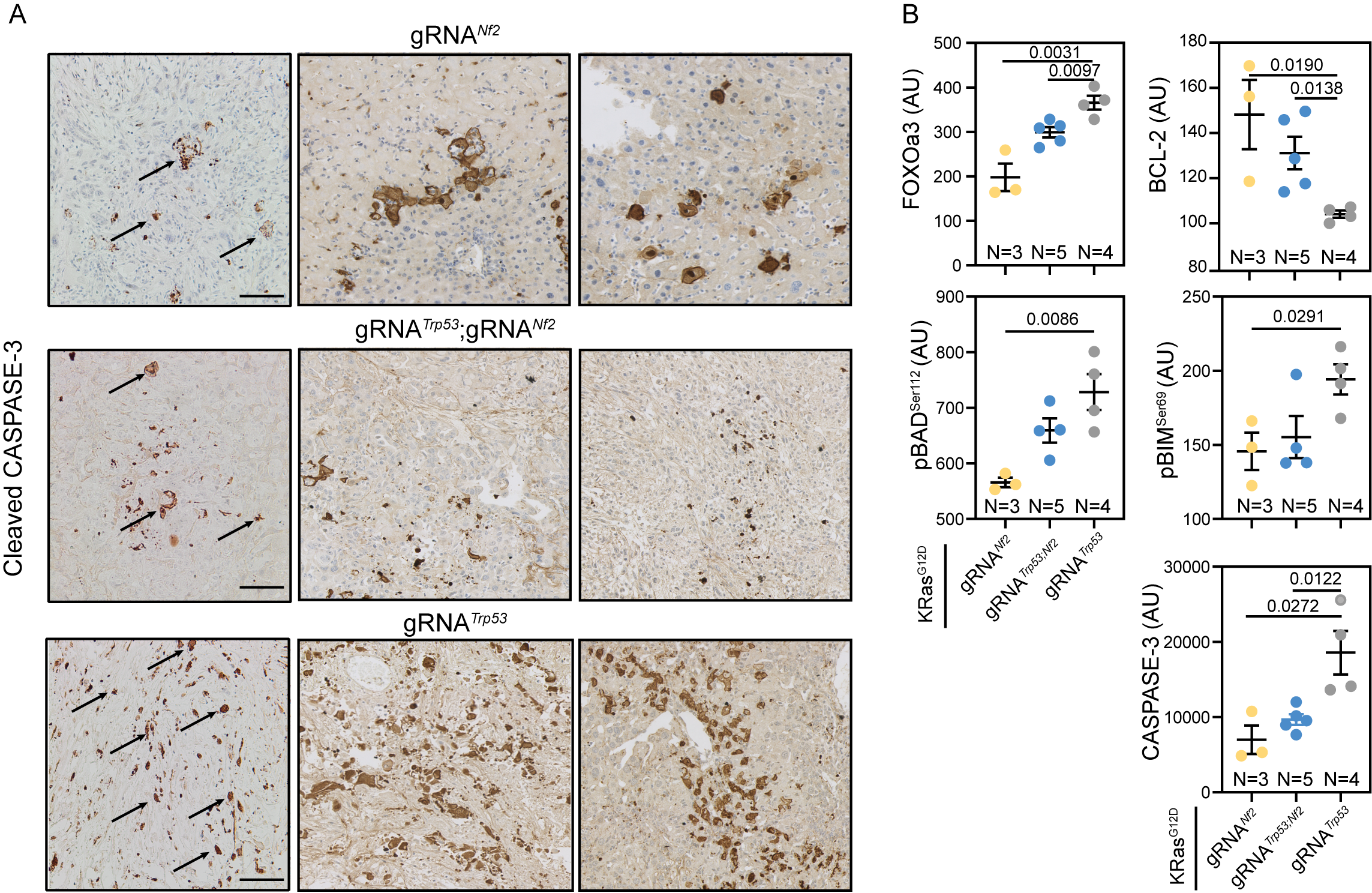


**Supplementary Figure 9 – The combined loss of *Nf2* and *Trp53* results in suppression of cancer cell apoptosis.** **A.** Immunohistochemistry staining for the apoptosis marker cleaved Caspase-3 on KRAS^G12D^;*Trp53*^KO^, KRAS^G12D^;*Nf2*^KO^, KRAS^G12D^;*Trp53*^KO^;*Nf2*^KO^ tissues. (Scale bar = 200 μm) **B.** RPPA analysis of apoptotic proteins using KRAS^G12D^;*Trp53*^KO^, KRAS^G12D^;*Nf2*^KO^, KRAS^G12D^;*Trp53*^KO^;*Nf2*^KO^ tumours as input material.


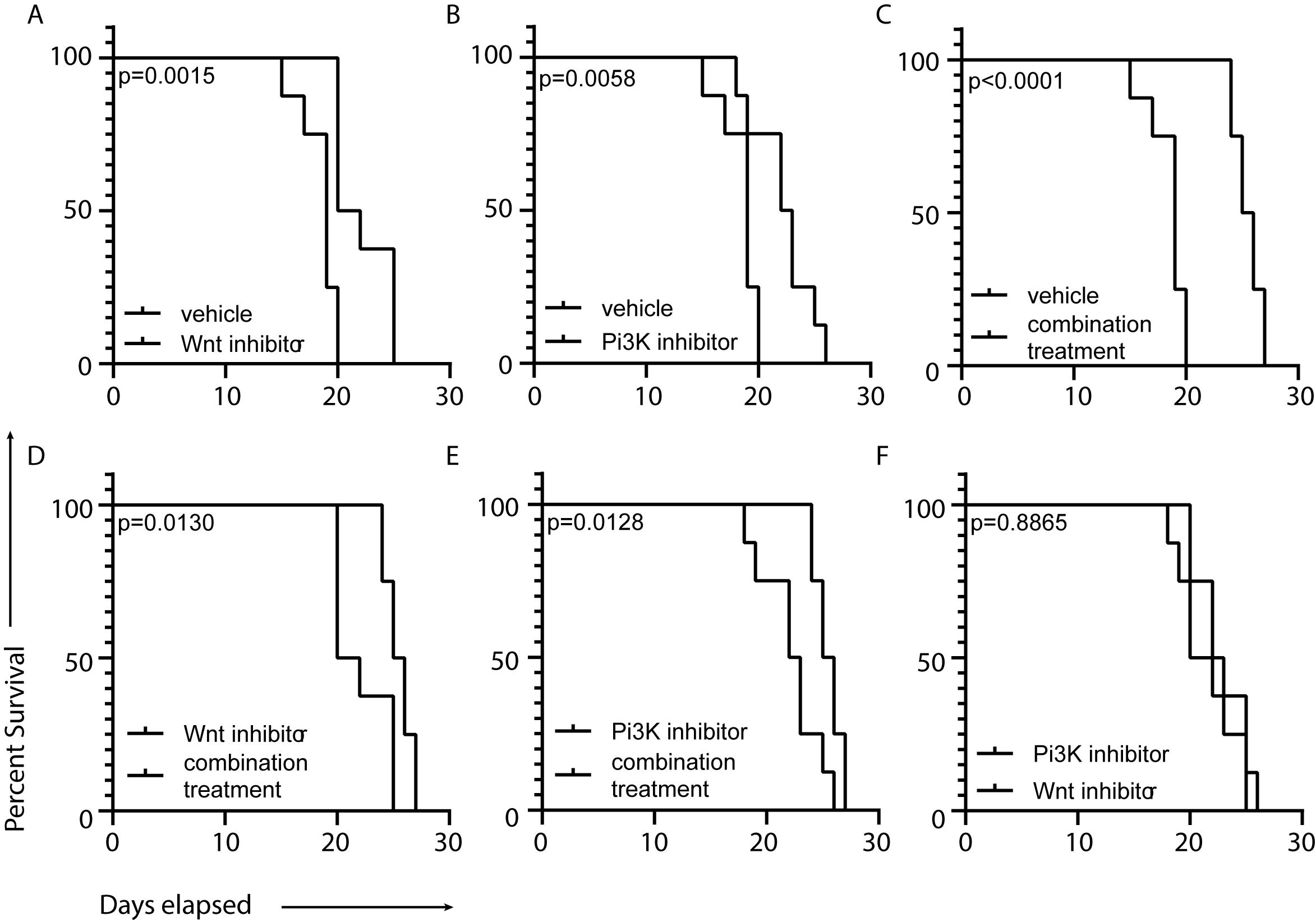


**Supplementary Figure 10 – Segregated survival data for animals treated with Wnt and PI3K inhibitors.** **A-F.** Kaplan-Meier curves of animals baring KRAS^G12D^;*Trp53*^KO^;*Nf2*^KO^ tumours treated with either vehicle, LGK974 (a Wnt inhibitor), Pictilisib (a PI3K inhibitor) or a combination of the two.
