# Supplementary figures and images for: In Vivo Modeling of Patient Genetic Heterogeneity Identifies New Ways to Target Cholangiocarcinoma
Source: Cancer Res. 2022 Jan 24;82(8):1548–59. doi: 10.1158/0008-5472.CAN-21-2556 (PMC9359731; doi:10.1158/0008-5472.CAN-21-2556)

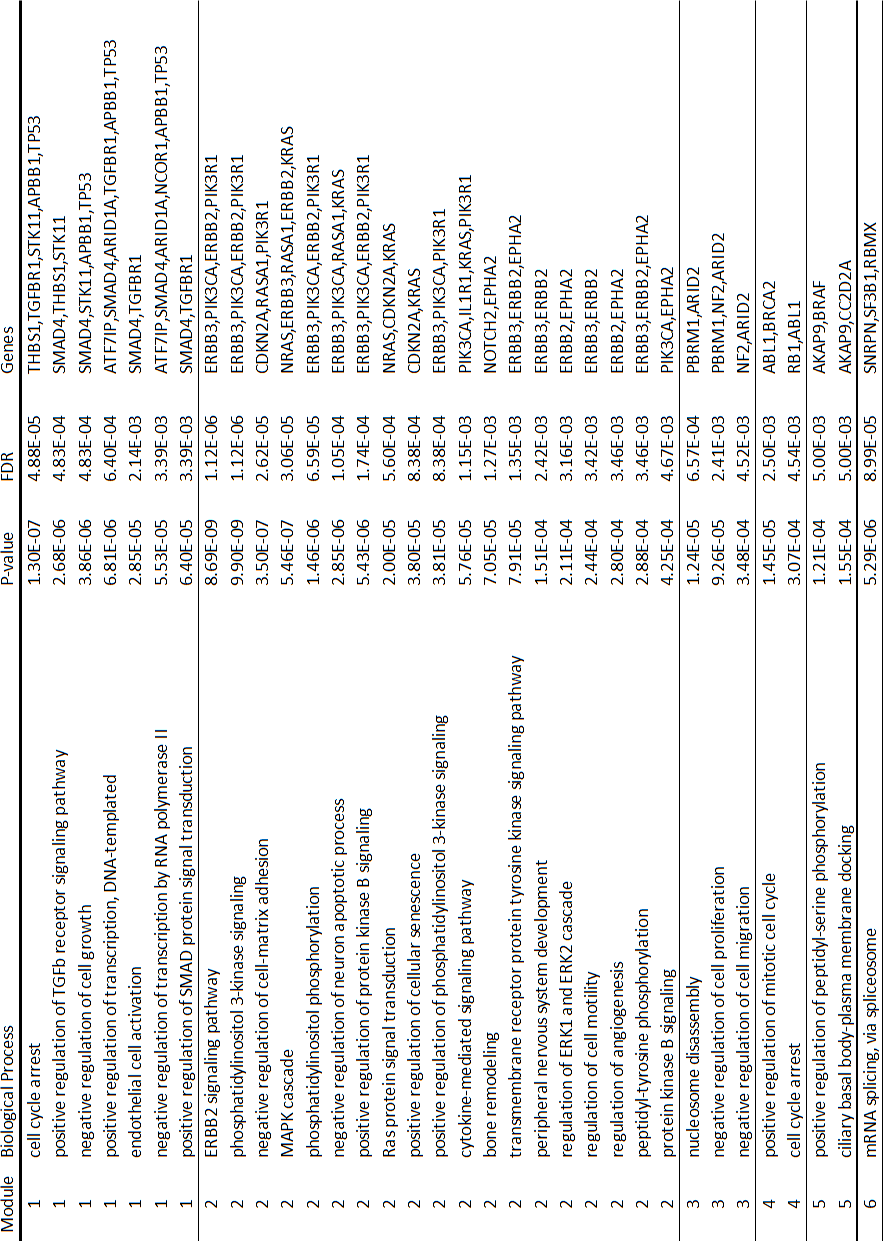


**Supplementary Table 6: Module Analysis of candidate driver genes found through IntOgen.**

Supplement: Supplementary Data [file can-21-2556_supplementary_data_suppst6.docx]
